# Fidelity of human ovarian cancer patient-derived xenografts in a partially humanized mouse model for preclinical testing of immunotherapies

**DOI:** 10.1136/jitc-2020-001237

**Published:** 2020-11-11

**Authors:** Adekunle Odunsi, A J Robert McGray, Anthony Miliotto, Yali Zhang, Jianming Wang, Adebukola Abiola, Cheryl Eppolito, Ruea-Yea Huang

**Affiliations:** 1 Center For Immunotherapy, Roswell Park Comprehensive Cancer Center, Buffalo, New York, USA; 2 Department of Gynecologic Oncology, Roswell Park Comprehensive Cancer Center, Buffalo, New York, USA; 3 Department of Biostatistics and Bioinformatics, Roswell Park Comprehensive Cancer Center, Buffalo, New York, USA

**Keywords:** immunomodulation, immunotherapy, adoptive, lymphocytes, tumor-infiltrating, tumor microenvironment, costimulatory and inhibitory T-cell receptors

## Abstract

**Background:**

Immune checkpoint blockers (ICBs) have been approved by the Food and Drug Administration to be used alone in front-line therapies or in combination with other regimens for certain advanced cancers. Since ICB only works in a subset of patients and has limited efficacy in treating ovarian cancer (OVC), developing preclinical models that help to understand which patients may derive benefit from ICB would be of tremendous benefit in OVC.

**Methods:**

Here, we generated preclinical human OVC models from freshly resected tumors, which include six patient-derived xenografts (PDXs) from six different patient tumors, three transplantable OVC PD spheroid lines (PD-sphs), and 3 cell lines (PD-CLs). We tested the therapeutic combination of anti-PD1/CTLA4 antibodies with (1) autologous tumor-associated leukocytes (TALs) on the growth of PD-sphs in a coculture system in vitro, (2) with adoptively transferred autologous peripheral blood mononuclear cells or TALs in patient-derived OVC models using partially humanized mice, NSG-HHDxSGM3 (N-HSGM3).

**Results:**

We show that PD-1 and CTLA-4 dual blockade when combined with autologous TALs effectively reduced PD-sph number in a co-culture system and led to regression of established PD-CLs and PDXs in the N-HSGM3 mice. Combinatorial PD-1 and CTLA-4 blockade increased the frequency and function of tumor-specific CD8 T cells. These CD8 T cells persisted in the tumor microenvironment, exhibited memory phenotype and protected animals from tumor growth on tumor rechallenge. Gene expression analysis of tumors resistant to dual PD1/CTLA4 blockade treatment identified upregulation of antigen processing and presentation pathways and downregulation of extracellular matrix organization genes.

**Conclusions:**

These findings describe a novel platform for developing patient-derived preclinical tumor models suitable for rationally testing combinatorial ICB in the context of autologous tumor-reactive T cells. This platform can be further developed for testing additional targeted therapies relevant to OVC.

## Introduction

Immune checkpoint blockers (ICB), specifically antibodies targeting the PD-1/PD-L1 and/or CTLA-4 pathways, have been shown to be effective in many advanced cancers.^1–3^ However, approximately one half of treated patients remain unresponsive to ICB therapies,^4^ and low response rates (~8% to 10% overall) to single agent ICB have been shown in ovarian cancer (OVC).^5 6^ Recent clinical trials have shown that ipilimumab (anti-CTLA4) plus nivolumab (anti-PD1) achieved higher response rate (~30%) than monotherapies in recurrent OVC.^7^ Conflicting data, however, have been reported using dual anti-CTLA-4 and anti-PD-1 blockade in the murine OVC model,^8–10^ possibly due to differences in preclinical tumor models and/or therapeutic antibody dosing schedules tested. These data indicate that the existing preclinical models exhibit poor predictive values for testing ICB combinations for OVC. Therefore, developing preclinical models for testing rationally defined immunotherapies in the context of cancer patient-derived tumors and immune cells may more accurately predict effective treatment strategies that can improve clinical outcome.

Patient-derived xenografts (PDXs) generated in immune compromised nonobese diabetic Cg-*Prkdc^scid^IL2rg^tm1Wjl^*/Sz (NSG) mice^7^ have been widely accepted as an important preclinical discovery platform for developing new avenues for personalized medicine. Conventional NSG mice carrying PDXs, however, are not appropriate for testing immunotherapies such as vaccines and ICB that act on immune cells. Humanized NSG mice reconstituted with CD34+ hematopoietic stem cells have been developed to study the efficacy of PD1 blockade on the growth of PDXs in several cancer types.^11^ However, potential drawbacks of the CD34+ humanized mouse model include a lengthy period (>12 weeks) for the engrafted CD34+ cells to fully differentiate into functional immune cells, and requirements for human leukocyte antigen (HLA) matching to avoid allogenic effects on the tumors^12^; a problem that can be prevented by using a completely autologous system.

Adoptive cell transfer (ACT) therapy using *ex vivo* expanded tumor infiltrating T cells has shown clear efficacy in treating melanoma and other tumors, including OVC,^13–16^ and is also effective in combination with anti-PD1 in treating melanoma PDXs in NSG mice.^17^ We have recently demonstrated that PDXs generated from an OVC patient’s tumor retained neoantigens inherited from the primary tumor and that these neoantigens can be recognized by autologous peripheral blood mononuclear cell (PBMC), which when infused into PDX tumor-bearing NSG mice could delay PDX-tumor growth.^18^ However, establishing PDXs has numerous inherent technical challenges, including (1) lengthy time required, (2) PDXs not efficiently established from all human tumors and (3) material availability limitations from a single patient as to generate sufficient PDXs for large-scale experimental testing. Patient-derived spheroids and organoids, in contrast, can be established faster and more efficiently, and have been shown to have great potential for studying tissue homeostasis and cancer.^19 20^ However, it is currently unclear whether these in vitro systems can adequately recapitulate the tumor microenvironment and, specifically, the immune landscape needed to effectively test candidate immunotherapies. Thus, developing a readily implementable and broadly employable in vivo patient-derived system to study cancer immunotherapies that (1) can identify rational combination approaches, (2) provide key mechanistic insights following treatment and (3) has the potential to predict clinical outcome and guide therapeutic decision making would be a tremendous asset for advancing promising preclinical approaches into the clinic.

Here, we sought to test the combination of ACT using human ovarian tumor-associated leukocytes (TALs) combined with ICB in a spheroid culture system and in PDXs utilizing a novel preclinical, partially humanized NSG mouse model. We designated the model NSG-HHD/SGM3 (N-HSGM3), wherein mice are heterozygous for both HHD II and SGM3 transgenes. The model HHD II is a transgenic HLA A∗0201 mouse that presents immunological epitopes through the HLA pathway.^21^ The NSG-SGM3 mice^22^ express 3 human transgenes (stem cell growth factor KITLG, granulocyte-macrophage colony-stimulating factor, and interleukin (IL)3) that allow superior engraftment of diverse hematopoietic lineages. Thus, this N-HSGM3 mouse model can be effectively used to investigate the role of both human T cells and myeloid cells in the antitumor immune response following infusion of cancer patient-derived TALs.

## Materials and methods

### Patient’s samples

Twenty-one tissue specimens, ascites fluids and 18 matched autologous PBMC were obtained with informed consent at Roswell Park Comprehensive Cancer Center (RP) in accordance with an approved protocol from the institutional review board. Clinical-pathological information for the entire cohort, including response to chemotherapy, is maintained in the Roswell Park (RP) Laboratory Information Management System database.

### Generating NSG-HHD/SGM3 mice

Three mouse strains were originally purchased from The Jackson Laboratory (Bar Harbor, Maine, USA) and bred in RP Laboratory Animal Resource under an approved IACUC protocol. NSG mice were used for establishing PDXs and as hosts for some tumor implantation. The N-HSGM3 strain was generated by crossing HLA-A2/HHD (HHD-II) mice with NSG-SGM3 mice to allow superior engraftment of diverse hematopoietic lineages.^23 24^ The presence of the four human transgenes in the N-HSGM3 was confirmed with quantitative PCR using TaqMan probes. The rate of mice developing graft-versus-host disease-like (GvHD-like) symptoms was estimated based on the number of mice showing symptoms of weight loss, ruffled fur or swollen legs after tumor implantation.

### Establishing PDXs, spheroids and cell lines

OVC tumor tissues were mechanically disrupted into aggregates of cells and passed through a size 100 µm mesh filter for establishing PDXs, spheroids and cell lines as detailed in online supplemental additional file 1, online supplemental methods.

10.1136/jitc-2020-001237.supp1 Supplementary data

### ACT and checkpoint blockade antibody treatment

Treatment schedules and doses are described in figures and figure legends, and in online supplemental methods.

### Flow cytometry and antibodies

Whole blood, spleen, ascites and tumor tissues were processed for flow cytometry analysis as described previously.^9^ Fluorescent-dye-conjugated antihuman antibodies are listed in online supplemental table 2.

### RNA sequencing and data analysis

Whole transcriptome sequencing was run on cells isolated from the peritoneal cavity washes or solid tumors isolated from untreated animals (phosphate bufferred saline, PBS) or animals treated with IgG or dual anti-PD1/CTLA4 blockade in combination with TALs. Detailed methods are described in online supplemental additional file 1, online supplemental methods.

## Statistical analysis

Results and statistical analyzes were generated with GraphPad Prism software. Summary statistics were calculated as mean±SE of the mean (SEM). Two-tailed, unpaired t-tests or the non-parametric Mann-Whitney U test were used to compare data from two treatment groups. One-way and two-way analysis of variances were used for data analysis of more than two groups. Statistical significance is indicated in each figure.

## Results

### Generation of OVC spheroids, PDXs and a humanized mouse model for testing OVC immunotherapies

**Figure 1:**
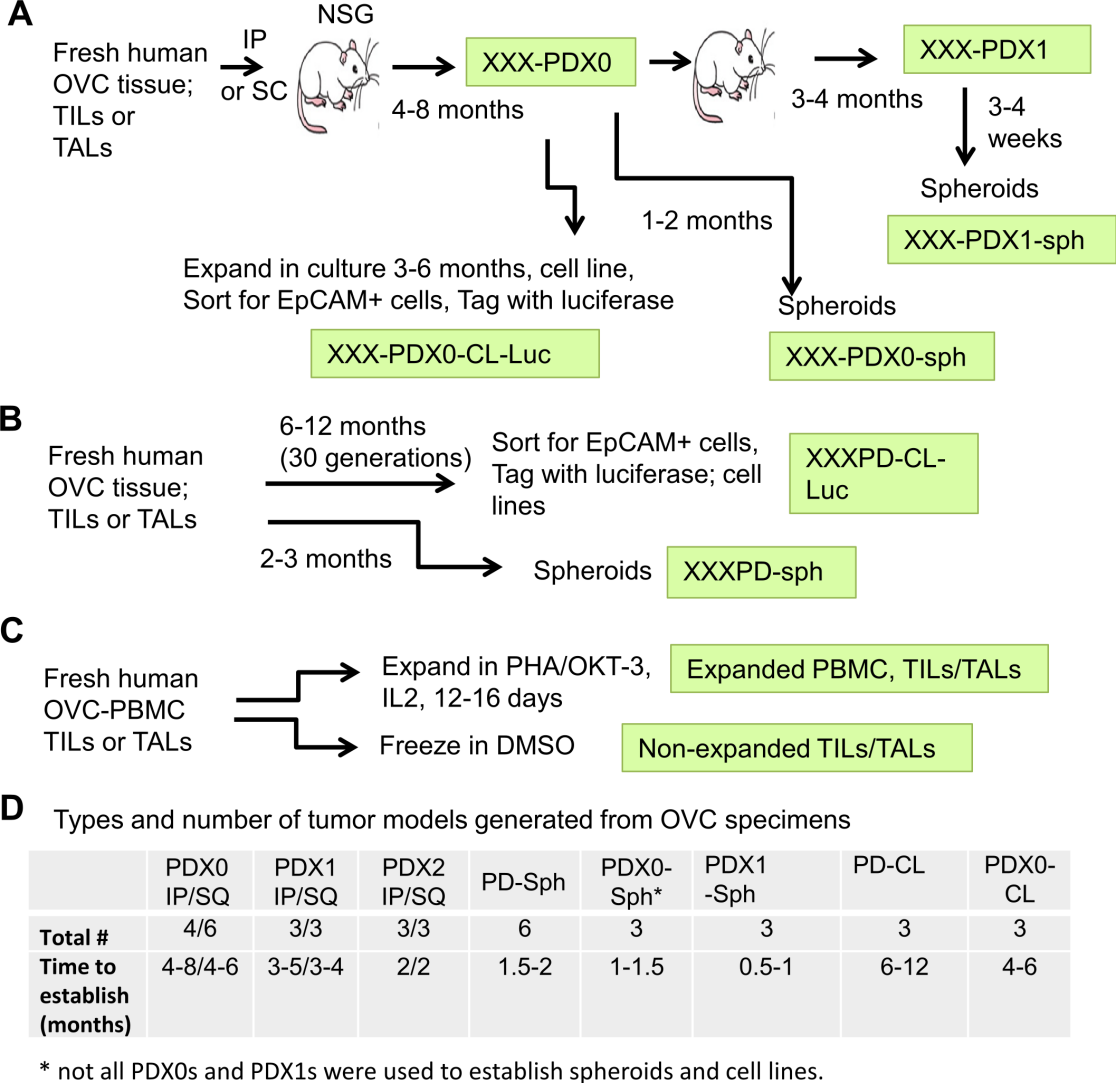
Overview of workflow for generating OVC PDXs in NSG mice, spheroids and cell lines, and expansion of TILs and TALs. (A) Establishment of PDXs. Fresh ovarian solid tumors or ascites fluid were made single cell or small clumps and injected intraperitoneally (IP) or subcutaneously (SQ) into NSG mice to generate PDXs. Tumors formed from the patients’ specimens were designated as PDX0. XXX represents tumor identification number. The PDX0 tumors were minced and reimplanted into tumor-naive NSG mice for next passage and were designated as Pdx1. Some of the PDXs were also expanded in vitro to generate spheroids (PDX0-sph) or cell lines (PDX0-CL) as described below. (B) Establishment of spheroids or cell lines. Cell suspension from the fresh human specimens or PDXs and their derivatives were cultured in spheroid growth medium or RMPI1640 complete medium to obtain spheroids (PD-sph) or cell lines (PD-CL), respectively. Some cell lines were sorted for epithelia phenotype with EpCAM antibody and tagged with luciferase for bioluminescence imaging. (C) Expanding autologous TILs/TALs. Single cells and/or small cell clumps were expanded using PHA (phytohaemagglutinin, 1 µg/mL) and OKT3 (1 µg/mL) in the presence of IL2 for 12–16 days or were frozen in 10% dimethyl sulfoxide (DMSO, non-expanded TILs/TALs). (D) Summary of types and number of tumor models generated from OVC specimens in this study. Average time observed in establishing the models is shown. IL, interleukin; IP, intraperitoneally; OVC, ovarian cancer; PBMC, peripheral blood mononuclear cell; PDX0-CL, patient-derived cell lines; PDX0-sph, patient-derived spheroid lines; SQ, subcutaneously; TALs, tumor-associated leukocytes; TIL, tumor-infiltrating leukocyte.

To generate targetable tumor models for preclinical ICB testing using autologous T cells, we used tissue specimens obtained from human OVC patients undergoing surgery which included PBMC, solid tumor tissue and ascites. Patient characteristics and histopathological factors in this study are shown in online supplemental table 1. The samples processed from the solid tumor tissue and ascites contained a mix of viable enriched immune and tumor cells, collectively called tumor-infiltrating leukocytes (TILs, from solid tumors) and tumor-associated leukocytes (TALs, from ascites) and 10 of these freshly isolated samples were used to establish PDXs in NSG mice subcutaneously (SQ) and/or intraperitoneally (IP) (figure 1A). The first generation of outgrowing tumors was designated as passage 0 (PDX0) and subsequently re-grafted tumors were designated as passage 1 (PDX1) then passage 2 (PDX2). In order to allow experimental comparison across multiple platforms, we also developed patient-derived spheroid lines (PD-sph and PDX-sph) and cell lines (PD-CL) in parallel using available tumor material (figure 1B). The number of PDX, PD-sph, PD-CL and PDX-CL established from 10 patient’s tumors is listed in figure 1D. Approximately 50% of the samples were able to successfully engraft as PDXs, which may be due to the differences in the aggressiveness of the patient tumors and ratio of tumor/immune cells in the samples at time of surgery and requires further investigation with additional samples. Exome sequencing analysis of 3 PDX0s from three different patient tumors indicated that the mutation loads of PDX0 were 80%–94% similar to that of the primary tumors (Want *et al*^18^ and unpublished data), suggesting that the PDX0s retained characteristics of the primary tumors. In addition, flow cytometry analysis of several different PDX0 indicated that immune cells were no longer present in the PDX0 samples (online supplemental figure S1). We have previously shown that the spheroids established from the OVC TILs/TALs (1) are able to form SQ and IP xenograft tumors (online supplemental figure S2), (2) have characteristics of serous adenocarcinomas of moderate/poor differentiation, similar to the parental patient’s tumors and (3) have similar architectural/cytological characteristics between the primary and grafted tumors.^25^ We successfully established PD-CLs from 3/10 patients (30%). PD-CLs were also engineered to express luciferase for in vivo bioluminescent imaging to monitor tumor progression. The autologous PBMC, TILs and TALs were also expanded using PHA (phytohaemagglutinin) and OKT3 in the presence of IL2 for 12–16 days (figure 1C) and banked for later use in ACT studies to test combinatorial ICB strategies.

10.1136/jitc-2020-001237.supp2 Supplementary data

### Clinical characteristics and immune composition of TILs/TALs from the human OVC patients

**Figure 2:**
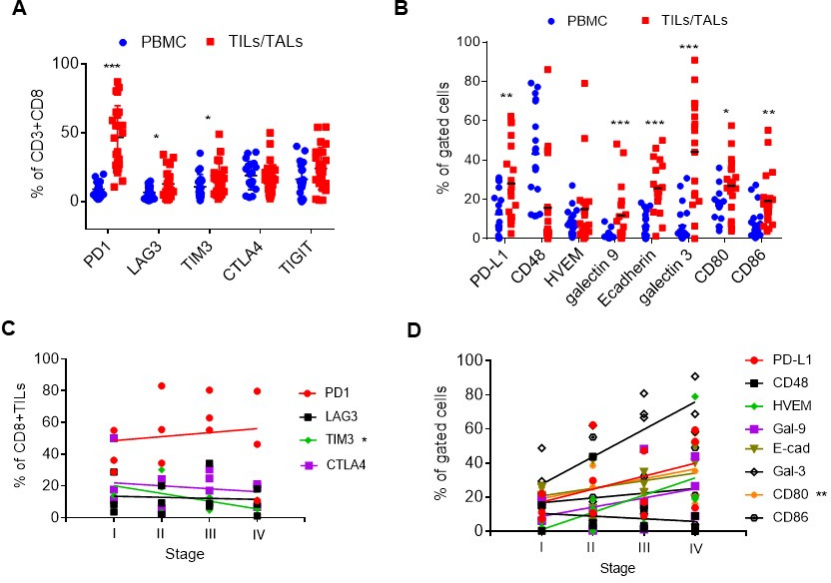
Expression of immune checkpoint receptors and ligands in the tumor samples of human OVC patients. (A) Expression of coinhibitory checkpoint molecules on the CD8 T+cell surface (PD-1, LAG3, TIM-3, CTLA-4 and TIGIT) in freshly thawed PBMC (blue dots) and TILs/TALs (red square). (B) Expression of checkpoint ligands in PBMC (blue dots) and EpCAM+ or EpCAM- (CD80, CD86) TILs/TALs (red square). Mann-Whitney U test: *P<0.05; **p<0.005; ***p<0.001. (C) The relationship between the level of coinhibitory checkpoint molecules on TILs/TALs and disease stages. Correlation, R^2^=0.91, *p<0.05. (D) The relationship between the level of cognate ligands in tumor samples and disease stages. Correlation, R^2^=0.99, **p<0.005. OVC, ovarian cancer; PBMC, peripheral blood mononuclear cell; TALs, tumor-associated leukocytes; TIL, tumor-infiltrating leukocyte.

To ensure that the relevant targets for the ICB chosen for testing in the patient-derived models were present on the TILs/TALs, the immune composition of 21 TILs/TALs was analyzed. All TILs/TALs were isolated and frozen at day of surgery and stained immediately after thawing for flow cytometry analysis. The clinical characteristics of these samples are summarized in online supplemental table 1). The levels of immune checkpoint (IC) receptors PD-1, CTLA-4, LAG-3, TIM3 and TIGIT in the CD8+ TILs/TALs were examined. As compared with 16 autologous and 3 non-autologous PBMC, the most highly expressed IC was PD-1, although others were also elevated (figure 2A; online supplemental figure S3A). To determine which of cognate ligands were also expressed at high levels in the OVC microenvironment as comparing to the peripheral blood, several ligands including those for the above receptors (PD-L1, CD48, galectin-9, HVEM (Herpes virus entry mediator), E-cadherin or galectin-3) were analyzed on epithelial cell adhesion molecule-positive (EpCAM+) tumor cells and ligands for CTLA4 (CD80, CD86) on EpCAM-negative cells (antigen presenting cells and stromal cells) in 19 TILs/TALs. Our analysis showed that cells found within the TILs/TALs also expressed higher levels of multiple ligands for the corresponding IC receptors as compared with cells in the PBMC (figure 2B; online supplemental figure S3B). These data support our previous findings that TILs/TALs in the TME of OVC patients exhibit phenotypes consistent with T cell exhaustion and that the isolated patient-derived materials would be suitable for establishing pre-clinical models designed to test the antitumor effects of targeting these inhibitory pathways.^26^ To investigate whether the expression level of the IC and their ligands in TILs correlate with clinical characteristics, a pilot study was conducted, where 12 TILs ranging from stage I to IV (online supplemental table 1) were selected for comparison. There was no clear correlation between the level of individual IC molecules on CD8+ TILs (figure 2C) and their ligands on EpCAM+ tumors/APCs (figure 2D) and clinical stage, possibly due to the small sample size used in the study. However, there was a significant negative correlation between the TIM3 level and disease stage (R^2^=0.91, p=0.04, figure 2C) and a significant positive correlation between the CD80 level and disease stages (R^2^=0.99, p=0.002, figure 2D). Based on the expression pattern of both receptor and ligand pairs and their clinical relevance, blockade of PD1 and CTLA4 pathways were chosen for subsequent studies using samples where 56%–80% of the original TILs/TALs expressed PD1, 33–61% expressed PD-L1, 35–50% expressed CTLA4 and 16%–58% expressed CD80.

### Autologous TALs in combination with checkpoint blockade can diminish the growth of OVC spheroids in culture

**Figure 3:**
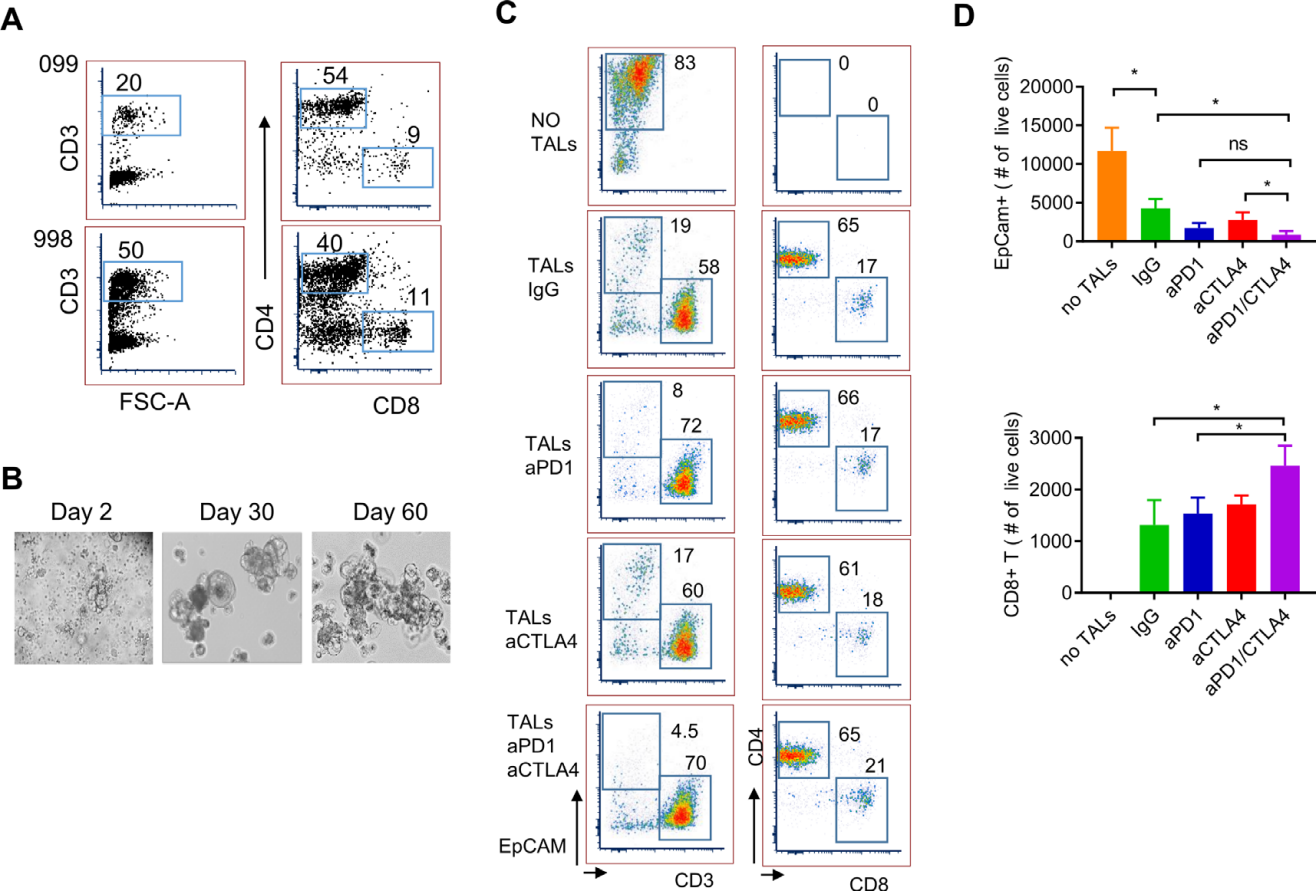
Checkpoint blockade in coculture of IL2-expanded TALs and patient-derived spheroids. (A) Frequency of CD3, CD4 and CD8 in IL2-expanded TALs from 998-OVC ascites samples. (B) Representative images of PD-spheroids (PD-sphs) in culture. Tumor-containing TILs/TALs were cultured in spheroid medium and images were taken at day 2, 30 and 60. (C) Flow cytometry analysis of ICB-treated PD-sphs and TALs in the presence of IL2. Representative flow plots of EpCAM+ tumor cells and T cells after ICB in coculture of PD-sphs and TALs. Numbers in the flow plots represent percentage of gated cell population. (D) Number of remaining live EpCAM+sph cells (top panel) and CD8+T cells (bottom panel). IL2-expanded autologous TALs (1×10^5^ cells) were mixed with 998PD-sphs (1×10^5^ cells, from day 60 culture) in spheroid growth medium and treated with IgG (16 µg/mL), anti-PD1 (15 µg/mL), anti-CTLA4 (16 µg/mL) or both anti-PD1 and anti-CTLA4 for 9 days in the presence of IL2 (100 U/mL). Medium with IL2 and antibodies were replaced at day 5. Cells were stained with antibodies for EpCAM, CD3, CD4 and CD8 populations. Data shown are mean±SEM of triplicate wells and are representative of duplicated experiments. Statistical significance is indicated as follows: *p<0.05 (Student’s t-test, two tailed and unpaired). ICB, immune checkpoint blocker; IL, interleukin; ns, not significant; OVC, ovarian cancer; PBMC, peripheral blood mononuclear cell; PD, patient-derived; SQ, subcutaneously; TALs, tumor-associated leukocytes; TIL, tumor-infiltrating leukocyte.

We first tested the effect of *ex vivo* expanded autologous TALs in combination with ICB antibodies on the growth of PD-spheroids (998PD-sph) in vitro. These *ex vivo* expanded TALs comprised 20%–50% CD3+ T cells and of these, 40%–50% were CD4+ T cells and 10%–20% CD8+ T cells (figure 3A). Figure 3B shows the morphology of the growing 998PD-sph at day 2–60. Spheroids (day-60) equivalent to 1×10^5^ cells were mixed with 1×10^5^ TALs in spheroid growth medium in the presence of IL2 with or without IgG, anti-PD1, anti-CTLA4 or both blocking antibodies for 9 days. The numbers of live EpCAM +tumor cells and CD8+ T cells were quantified using flow cytometry. Autologous TALs alone were able to reduce the number of EpCAM+ cells while both anti-PD1 and anti-CTLA4 monotherapies further decreased the population of EpCAM+ cells down to 20%–25% of untreated (figure 3C left column; figure 3D top panel, online supplemental figure S4A). Dual blockade with anti-PD1/CTLA4 significantly reduced EpCAM+ cell number (10% of IgG control). The absolute number of CD8+ T cells was significantly higher in the dual ICB treated samples as compared with that of the IgG and anti-PD1 treatment (figure 3D bottom panel), while the percentages of both CD8+ and CD4+ TALs remaining in the cultures were similar among all treatment groups (figure 3C right column, online supplemental figure S4A). Similar results were observed in coculture of 099PDX0-sph and expanded autologous TALs (online supplemental figure S4B). Furthermore, addition of blocking pan anti-major histocompatibility complex (MHC) class I (HLA-A, B and C) antibody (40 µg/mL) significantly increase the number of the EpCAM+ cells as compared with the isotype control (online supplemental figure S5), suggesting specific tumor recognition and killing by CD8 +T cells. The incomplete effect of blocking the HLA class I implicated the likely involvement of CD4+ T cells/HLA class II pathway in this system. Nonetheless, these data provide evidence that patient-derived ovarian tumor spheroids can be used in coculture with autologous tumor-derived T cells for testing the effectiveness of checkpoint blockade reagents on antitumor immunity.

### Combinatorial PD1 and CTLA4 blockade with adoptively transferred autologous PBMCs inhibit OVC growth in vivo in NSG mouse models

**Figure 4:**
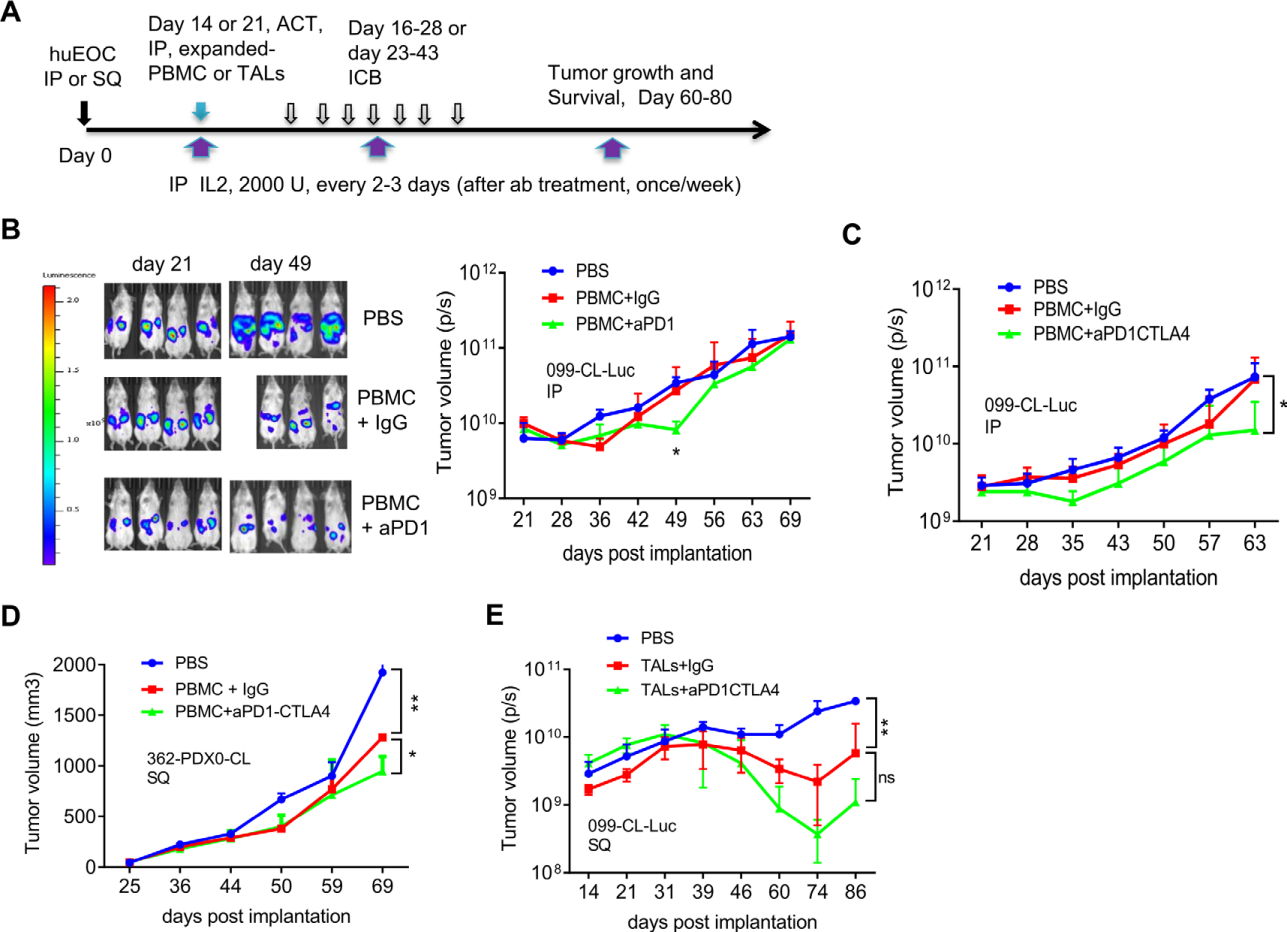
Checkpoint blockade combined with adoptively transferred autologous PBMC or TALs slowed the OVC growth in NSG mice. (A) Experimental schema for tumor implantation, adoptive cell transfer (ACT) and antibody blockade treatment. tumor cells were IP or SQ injected into NSG mice. Twenty-one (or 14 in E) days after tumor implantation, mice were treated (IP) with autologous PBMCs or TALs and IL2 (2000 U). IgG or ICB antibodies (anti-PD1, 200 µg/mouse; anti-CTLA4, 100 µg/mouse), and IL2 were IP starting day 16 or 23 every 2–3 days for seven times. Thereafter mice were treated with IL2 once per week. Tumor volume was assessed by bioluminescent imaging (P/S, photons/s in B, C and E) or caliper measurement (D) of tumor-bearing mice at indicated time points post implantation. (B) Adoptive transfer of IL2-expanded autologous PBMC (1×10^6^) in combination with anti-PD-1 blockade mildly delayed the growth of 099-luc-CL (IP) in NSG mice (n=3–4). (C) Adoptive transfer of IL2-expanded autologous PBMC (1×10^6^), in combination with anti-PD-1/CTLA4 blockade significantly delayed the growth of 099-luc-CL (IP) in NSG mice (n=3). (D) Adoptive transfer of autologous PBMC (1×10^6^) in combination with anti-PD-1/CTLA4 blockade significantly delay the progression of 362-PDX0-CL (SQ) in NSG mice (n=2–3). Tumor volume data were determined according to formula: (length × wide^2^)/2. (E) Adoptive transfer of IL2-expanded autologous TALs in combination with anti-PD-1/CTLA4 dual blockade significantly delayed the growth of 099-CL-Luc (SQ) in N-HSGM3 mice (n=3). Autologous TALs (4×10^5^) were IP-injected at day 14 and IgG or CPB starting at day 16 for seven times. Tumor volume was assessed by bioluminescent imaging (protons/sec) since the tumor express luciferase at indicated time points post implantation. Tumor volume data were from mice reach end points and are presented as mean±SEM from one of the duplicated experiments. *P<0.05, **p<0.01, ns, not significant, using multiple t-test (B) or two-way ANOVA (C, D, E). ANOVA, analysis of variance; IL, interleukin; IP, intraperitoneally; OVC, ovarian cancer; PBMC, peripheral blood mononuclear cell; SQ, subcutaneously; TALs, tumor-associated leukocytes; TILs, tumor-infiltrating leukocytes.

We previously demonstrated that PBMCs and TILs/TALs from OVC patients harbor tumor-recognizing T cells, and have efficacy toward autologous tumors.^25–28^ We asked whether adoptive transfer of OVC patient-derived autologous PBMCs or autologous TILs/TALs in combination with ICB can control OVC growth in NSG mice. The general scheme of tumor implantation, ACT, IL2 treatment and checkpoint blockade schedules are depicted in figure 4A. We first tested the effect of combining adoptive transfer of autologous PBMCs with PD-1 blockade on the growth of 099-CL-Luc which had been engineered to express luciferase. Twenty-one days after IP tumor implantation, tumor growth was measured using bioluminescence imaging (figure 4B). Mice were treated with PBMC, anti-PD-1 or IgG control antibodies and IL2. PD1 blockade slowed the growth of 099-CL-Luc tumors in NSG mice (figure 4B), however, the effect was not sustained and suggested limited improvement in the durability of T cell attack following single agent anti-PD-1. Based on these data, the *in vitro* blocking studies using patient-derived spheroids (figure 2C), as well as our previous murine OVC study demonstrating that only dual anti-PD1/CTLA4 blockade significantly reduced OVC growth,^13^ we next focused on a dual anti-PD1/CTLA4 blockade strategy, which was used in all subsequent studies. To this end, we tested combining autologous PBMCs with dual PD-1/CTLA4 blockade in two xenografts, 099-CL-Luc (figure 4C) and 362-PDX0-CL (figure 4D), which were derived from different patients. The latter was implanted SQ to allow careful serial monitoring of tumor burden and to test therapeutic efficacy in another tumor site. Strikingly, adoptive transfer of IL2-expanded autologous PBMC in combination with anti-PD-1/CTLA4 blockade, significantly delayed the growth of both 099-CL-Luc (figure 4C, IP tumors) and 362-PDX0-CL (figure 4D, SQ tumors). Together, these studies demonstrated in two different tumor models that IL2-expanded autologous PBMC could recognize and kill tumor cells in vivo and the effect was enhanced by dual PD-1/CTLA4 blockade, but that tumor control was not durable.

N-HSGM3 mice exhibit reduced GvHD and allows persistence of adoptively transferred autologous TALs to efficiently control OVC progression when combined with dual anti-PD1 and anti-CTLA4 blockade.

**Figure 5:**
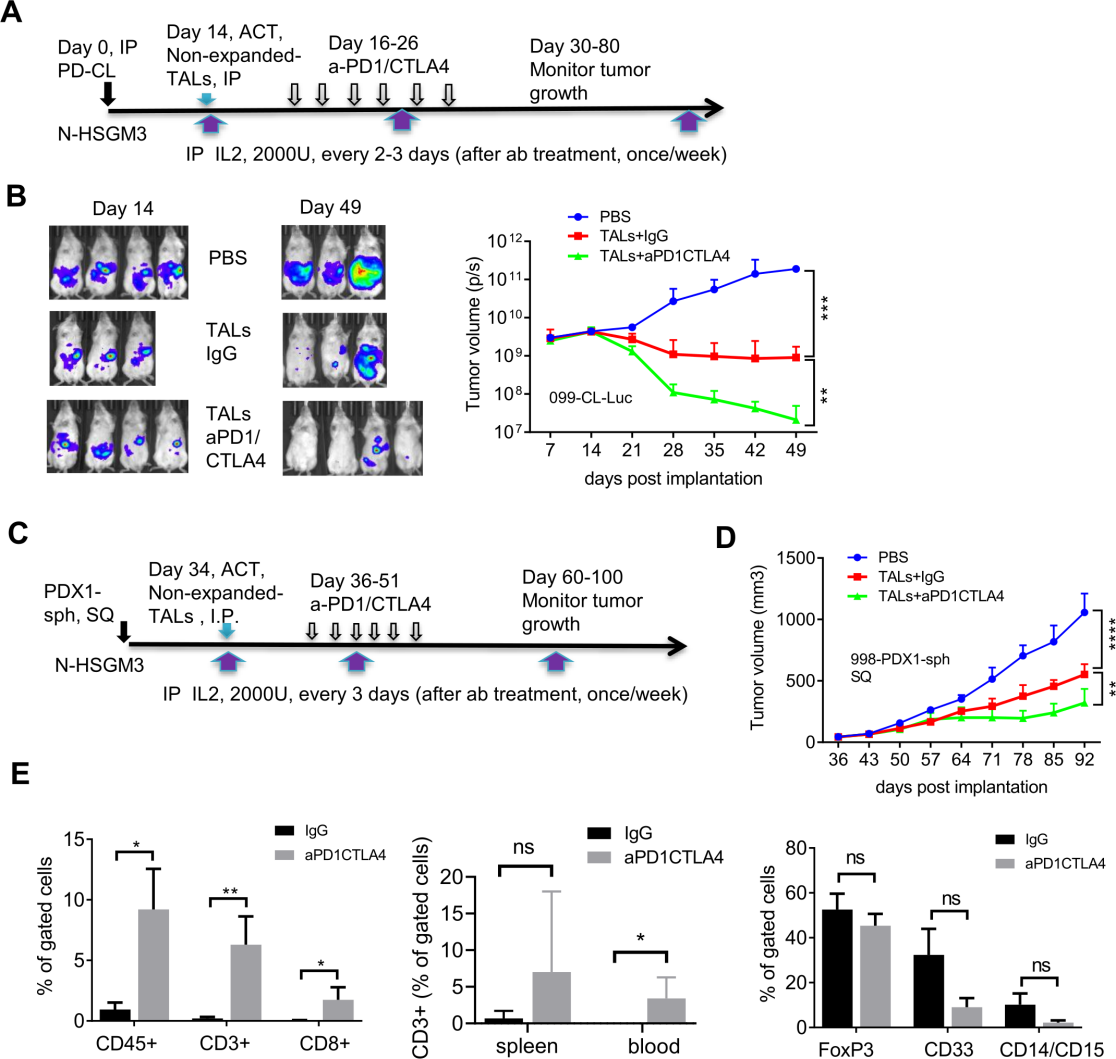
Adoptive transfer of non-expanded autologous TALs in combination with PD-1/CTLA4 blockade significantly inhibit the growth of IP-injected 099-CL-Luc and SQ-injected 998-PDX1-sph in N-HSGM3 mice. (A) Schema of 099-CL-Luc tumor implantation, ACT and ICB treatment. Fourteen days after tumor implantation, mice (n=3–4) were imaged and treated intraperitoneally with IgG or blockade antibodies every other day for six times. (B) Tumor load was assessed by bioluminescent imaging of 099-CL-Luc OVC tumor-bearing mice at indicated time points post implantation. Tumor volume data are presented as mean±SEM, and are from one of the duplicated experiments. (C) Schema of 998-PDX1-sph tumor implantation, ACT and ICB treatment. (D) Tumor growth of 998-PDX1-sph was assessed using a caliper at indicated time points postimplantation. Tumor volume data are presented as mean±SEM from one of the two independent experiments. (E) Dual ICB increased CD3+CD8+ T cells and decreased CD33+ MDSC cells in the peritoneal wash (PW) Tme. Left panel, frequencies of CD45+, CD3+ and CD8+ T cells were all significantly higher in the aPD1/CTLA4-treated PW than the IgG-treated group. Middle panel, frequency of CD3+ T cells in the spleen and blood samples. Right panel, frequency of FOXP3 and MDSC populations in PW samples. Live cells from the peritoneal wash, spleen and blood samples from the IgG- and dual aPD1/CTLA4-treated mice were stained with antibodies specific for CD45, CD3, and CD8 for T cells or CD4, CD25, and FOXP3 for T regulatory cells, or CD45, CD33, CD11b, CD14, and CD15 for MDSC cells. *P<0.05, **p<0.01, ***p<0.001, ****p<0.0001, ns, not significant, using two-way ANOVA (B, D) or unpaired two-tail t-test (E). ACT, adoptive cell transfer; ANOVA, analysis of variance; ICB, immune checkpoint blockers; IL, interleukin; IP, intraperitoneally; MDSC, myeloid-derived suppressive cells; OVC, ovarian cancer; PBMC, peripheral blood mononuclear cell; SQ, subcutaneously; TALs, tumor-associated leucocytes; TILs, tumor-infiltrating leukocytes.

We speculated two possibilities that might prevent durable tumor control when combining ACT of PBMC and dual PD1/CTLA4 blockade despite continual delivery of IL2 throughout the study: (1) low frequency of tumor-specific T cells in the PBMC, (2) the life-span or quality of transferred T cells in the NSG mice. To increase the number of tumor-specific T cells, we used patient’s TALs for ACT, which generally yield a greater frequency of viable T cells after activation than could be obtained from TILs.^26^ The N-HSGM3 strain was used to circumvent the second possibility (poor T cell persistence). While the frequency of the CD3, CD8 and CD4 cells in the blood/spleen are similar among the NSGHHD-II and NSGSGM3 mice following ACT (online supplemental figure S6A-B, data not shown), there were more CD8 cells in N-HSGM3 mice compared with NSG mice at day 60 after infusion of HLA2-A2+normal PBMCs (online supplemental figure S6A). Furthermore, the engraftment and growth of the HLA2-A2+099-CL-Luc cells without treatment were similar between NSG and N-HSGM3 strains (online supplemental figure S6C). Importantly, a fraction of the tumor-bearing NSG mice developed GvHD-like symptoms beginning around 6 weeks post tumor implant (30%–40%); this resulted in early termination of experiments or reduced sample sizes, thus limiting the utility of the NSG strain for our purposes. In contrast, the rate of GvHD symptoms was reduced in N-HSGM3 strain (~10%). Hence, we tested the therapeutic effect of IL2-expanded autologous TALs alone or in combination with anti-PD-1/CTLA4 blockade in N-HSGM3 mice and found that autologous TALs alone significantly inhibited the growth of 099-CL-Luc tumors while TALs plus ICB further delayed the growth of SQ 099-CL-Luc (figure 4E). Although ACT of autologous TALs in combination with dual ICB blockade prolonged survival of the N-HSGM3 mice, tumors were never completely cleared and the T cells were no longer present in the blood by day 60 post transfer in most of the mice or in the TME at endpoint (day 70–86, data not shown). We speculated that the *ex vivo*-expanded TALs already expressed IC receptors at high levels due to activation and became exhausted quickly following ACT. Indeed, flow cytometry analysis showed that these IL2-expanded TALs expressed high levels of multiple checkpoint proteins after *in vitro* expansion (online supplemental figure S7A), which may lead to rapid T cell exhaustion after transfer despite ICB treatment. We reasoned that non-expanded autologous TALs might be more effective than the IL2-expanded T cells since the levels of checkpoint proteins were lower prior to *in vitro* expansion (online supplemental figure S7B). Furthermore, an additional advantage of using non-expanded TALs for ACT was that these TALs comprise the complete immune landscape of the excised tumor, including myeloid cells (macrophages, dendritic cells, myeloid-derived suppressive cells (MDSC), (online supplemental figure S7C) and regulatory T (Tregs), and may better recapitulate the natural immune landscape of the patient’s tumor. In this regard, the N-HSGM3 strain is advantageous as it carries three human transgenes that better support engraftment of diverse hematopoietic cells. In line with this, we observed that the frequency of different MDSC populations in the peritoneal wash (PW) trended higher in tumor-bearing N-HSGM3 compared with NSG mice at day 30 postinfusion of non-expanded TALs (online supplemental figure S8). Thus, we infused non-expanded autologous TALs along with weekly IL2 support into 099-CL-Luc tumor-bearing N-HSGM3 mice and tested therapeutic efficacy either alone or in combination with ICB (figure 5A). As shown in figure 5B, TALs alone had significant efficacy in controlling IP tumor growth. Most interestingly, half of the mice in the group with anti-PD-1/CTLA4 dual blockade did not have detectable luciferase signal at day 49, suggesting complete tumor regression following the combination therapy. We further confirmed this finding in the 998-PDX1-sph model, a PDX1-derived spheroid line (described in figure 1). When the spheroids equivalent of 2–3 x 10^5^ cells were implanted SQ, 998-PDX1-sph formed tumors (approximately 40–50 mm^3^) at day 30 in N-HSGM3 mice. figure 5C, D demonstrated that non-expanded autologous TALs were able to significantly delay 998-PDX1-sph tumor growth in mice as compared with the non-treatment group. Dual blockade with anti-PD1/CTLA4 antibodies further improved tumor control by the autologous TALs, though the response was less robust and of shorter duration than that observed for 099-CL-Luc tumors (figure 5B).

### Dual anti-PD1/CTLA4 blockade promotes the function and persistence of the transferred non-expanded autologous TALs

To further test the antitumor function of the TALs that persisted long-term following complete or near complete response to dual ICB, we rechallenged three mice with very low or no luciferase signal at day 56 postinitial tumor implantation. Notably, these mice still had detectable circulating CD3+ cells in the blood at the time of rechallenge (data not shown). As a control for IP injection we also implanted three tumor-naive mice with the same number of tumor cells. The rechallenged mice were also treated with additional 6 doses of anti-PD1 and anti-CTLA4 antibodies. Online supplemental figure S9A shows that the residual TALs in 2/3 mice were able to control the re-implanted 099-CL-Luc tumor for more than 50 days after the control mice reached end point. After this point, tumors in the rechallenged mice progressed and no immune cells were detectable at experimental end point (90 days post-tumor rechallenge), suggesting a limited duration of T cell persistence in this model may ultimately prevent long-term tumor control. We next investigated the status of the infused non-expanded autologous TALs in the TME to test whether ICB reinvigorated cytotoxic T cells thereby enhancing antitumor immunity. As ICB treated tumors has almost completely regressed at the time of analysis, we compared immune cells in PW samples at day 53 post-tumor implantation. Blood and spleen were also assessed for any systemic trafficking of the transferred TALs. In the TME, the dual anti-PD1/CTLA4 blockade group had approximately 10 times the frequency of CD45+ cells, and 30 times the frequency of CD3+ and CD8+ T cells as compared with the IgG controls (figure 5E, left panel; online supplemental figure S9B). Intriguingly, dual anti-PD1/CTLA4 blockade treatment group also had a modest increase of CD3+ cells in the blood and spleen (figure 5E, middle panel), suggesting that the infused T cells trafficked out of the TME following complete tumor clearance. In contrast, dual anti-PD1/CTLA4 blockade did not affect the frequency of the immunosuppressive CD4^+^CD25^+^FoxP3^+^ Treg cell populations (figure 5E, right panel). Finally, we also evaluated the MDSC populations in the TME. As shown, dual anti-PD1/CTLA4 blockade resulted in reduced CD33 +myeloid cells and CD14+CD15+MDSCs when compared with the IgG-treated group (figure 5E, right panel). We also observed that total CD56+NK or NK-T cells constituted approximately 40%–50% of the remaining CD45+ population regardless of the treatments (online supplemental figure S9C). Although dual ICB blockade significantly enhanced the impact of ACT on 998-PDX1-sph tumor growth, it did not increase the frequency of CD8 T+cell or decrease the MDSC population at study endpoint (online supplemental figure S10).

### Acquired resistance to dual PD1/CTLA4 blockade is associated with transcriptional changes in antigen processing pathways and extracellular matrix organization

**Figure 6:**
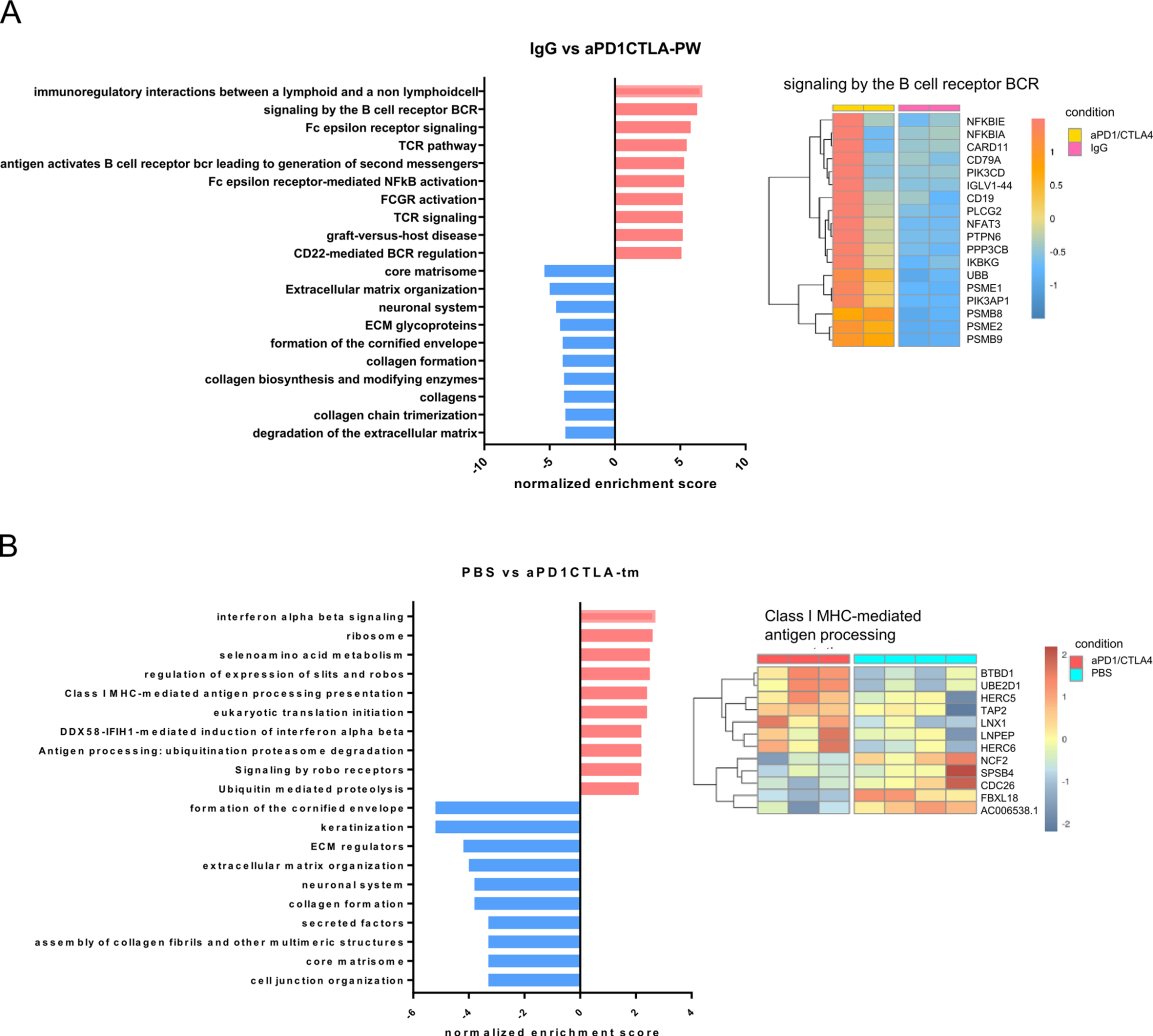
GSEA showing the top 10 enriched pathways responded to dual anti-PD1/CTLA4 blockade. (A) Bar plots showing the Normalized Enriched Score (NES) of the top 10 upregulated (red) and downregulated (blue) pathways in the peritoneal wash samples from mice treated with dual anti-PD1/CTLA4 blockade relative to IgG control. Representative heatmap showing one of the upregulated pathways (right panel). (B) Bar plots showing the NES of the top 10 upregulated (red) and downregulated (blue) pathways in the tumor samples from mice treated with TALs plus anti-PD1/CTLA4 blockade relative to PBS control. Representative heatmap showing one of the upregulated pathways (right panel). GSEA, gene set enrichment analysis; TALs, tumor-associated leukocytes.

To understand additional mechanisms of resistance to ICB intervention and the potential utility of the humanized model to provide novel insights regarding tumor immune escape, we performed transcriptome profiling by RNA-sequencing (RNA-seq) of PW samples from mice that responded to aPD1/CTLA4 along with IgG controls (day 53 in figure 5B), solid tumors from treated mice that relapsed after re-challenge (online supplemental figure S6C), as well as control animals treated with PBS (figure 5B). Differential gene expression and gene set enrichment analysis (GSEA) were performed using gene sets with greater than or equal to twofold change at FDR<0.05 between groups. GSEA from the PW samples (anti-PD1/CTLA4 vs IgG) showed that dual ICB blockade significantly increased expression of pathways involved in immuno-regulatory and TCR/BCR signaling, while downregulating pathways associated with extracellular matrix organization, collagen formation and glycosylation (figure 6A). These data correlated well with the findings that dual blockade increased the frequency of CD3/CD8 T cells in the TME (figure 5E) and further suggested that reduced expression of matrix remodeling genes occurs in regressing tumors. Representative heatmaps of the top 10 enriched pathways are shown in (figure 6A and online supplemental figure S11A). We next interrogated transcriptional changes between solid tumors from mice which relapsed following TALs+aPD1CTLA4 treatment (online supplemental figure S6C) and from untreated mice (PBS, figure 5B). GSEA revealed significant upregulation of pathways involved in interferon-alpha/beta signaling and class I MHC/ubiquitination-mediated antigen processing in relapsed tumors following TALs+ aPD1/CTLA4 treatment while processes including in keratinization and collagen formation, and extracellular matrix remodeling were downregulated (figure 6B, online supplemental figure S11B). Taking together, these data reveal that anti-PD-1/CTLA4 treatment modulates complex transcriptional changes in both infused TALs and tumors and that the balance of enrichment between immune/inflammatory pathways and matrix remodeling/genome integrity pathways have important implications in driving treatment outcomes.

## Discussion

Preclinical studies can play a critical role in providing insightful information to clinicians to launch appropriate clinical trials that are safe and likely to be effective for a particular tumor indication and patient population. However, many initially promising therapies identified using existing preclinical models have failed to achieve this goal.^29 30^ In the case of current ICB strategies, there is a clear disconnect between preclinical efficacy demonstrated in mouse models and clinical outcome observed in OVC patients.^8–10^ Toward the goal of reducing this disparity, we have developed improved preclinical OVC models utilizing patient-derived material as a means of testing combination ICB in a context that more accurately reflects human OVC. As a proof of concept, we tested ICB in the setting of adoptively transferred autologous PBMC or TALs targeting autologous patient-derived OVC models using a novel partially-humanized N-HSGM3 mouse model. We have also demonstrated the potential of targeting patient-derived spheroids (PD-sphs) by ICB by coculturing them with autologous TALs. While this in vitro model undoubtedly lacks some TME-intrinsic attributes that are more readily assessed using *in vivo* models, our findings support the parallel development of *in vitro* approaches, which may prove particularly valuable in settings where limited clinical material is available (and thus precludes *in vivo* testing) or in instances where *in vivo* PDX establishment is unsuccessful.

Improved PDXs and mouse models have been recently designed for testing chemotherapies and immunotherapies.^17 31–34^ One of the major advances made using our preclinical platform is the parallel establishment of multiple experimental materials from precious patient’s specimen, encompassing autologous PD-CL and PDXs that engraft efficiently with similar rates among mice and viable TALs that can be infused and remain responsive to ICB in the context of autologous tumor. We have also demonstrated that PDX-sphs from two different patients can be used together with autologous T cells for *in vitro* preclinical ICB testing. However, it will be important in future studies to compare response rates using different type of models developed from the same patients and with those having varying disease status/treatment histories to further assert whether different approaches to developing preclinical models have superior utility. Another strength of the current model is the use of the partiallyhumanized N-HSGM3 mice that are designed to improve the engraftment of infused immune cells and reduce GvHD, thereby establishing a more suitable tumor-immune contexture for testing immunotherapy strategies. In line with this, our data clearly demonstrated that dual anti-PD1/CTLA4 blockade enhances the frequency and persistence of the non-expanded autologous TALs in controlling the progression of PD-CL or PDX-sph in the N-HSGM3 mice.

One of the important objectives of cancer immunotherapy is to elicit a sustained tumor-specific T cell response. Here we have demonstrated that the durability of the antitumor response exhibited by the anti-PD1/CTLA4 combinatorial treatment is consistently greater than that of the IgG controls following ACT using either autologous PBMC or TALs (figure 4). The efficacy of the autologous PBMC is surprizing, since it has low frequency of the tumor reactive T cells,^35^ but is consistent with our recent finding.^18^ Our data clearly demonstrated that the non-expanded autologous TALs are suitable for preclinical ACT testing based on (1) improved tumor control, (2) the frequency of T cells infiltrating into the TME and (3) the persistence and durable tumor attack by TALs in the rechallenged mice. These findings are consistent with our previous findings in a syngeneic C57BL/6 mouse OVC model,^13^ where dual anti-PD1/CTLA4 blockade led to more durable and functional TILs/TALs and improved tumor control. The observation that dual ICB in combination with non-expanded autologous TALs produced more durable tumor control than with IL2-expanded autologous TALs may be due to the possibilities that (1) the *in vitro* TAL expansion leads to preferential expansion of highly proliferative bystander T cells,^36 37^ (2) the non-expanded TALs may have better cellular fitness judging from the level of exhaustion markers on their surface while the *ex vivo* expanded T cells are more likely to be terminal differentiated effector T cells that may become exhausted quickly^38^ and (3) the presence of APC in the non-expanded TALs may improve T-cell priming, provide additional costimulatory signals to T cells,^39^ and cooperate with ICB treatment.^40^ We cannot rule out the possibility that the discrepancy may be due to trafficking of the transferred TALs to different tumor sites since the expanded TALs had to traffic from the IP cavity to the SQ tumor site. This warrants future investigation by direct comparison of IP and SQ tumor models from same PDXs. However, as therapeutic efficacy of non-expanded TALs/ICB combination was seen in both 099-CL-Luc in a IP tumor model (figure 5B) and 998-PDXsph in a SQ tumor model (figure 5C–D), this suggests that non-expanded autologous TALs are well-suited donor source for ACT in preclinical testing for OVC. The major drawback of using the non-expanded TALs is that the source of the patient material and the amount of TALs available may be limited. We believe that enhancing the strength and number of the infused T cells is required to prolong and increase the efficacy of ACT.

The PDX and the N-HSGM3 models together with the checkpoint blockade tested here have the potential to identify factors leading to resistance to ICB treatment. Our RNA-seq results have uncovered differential regulation of pathways in tumors that are originally responsive to and later become resistant to ACT/ICB treatment. Of particular interest are the class I MHC-mediated and ubiquitination-mediated antigen processing pathways. While loss of beta-2 microglobulin, a membrane protein component of the MHC class I has been associated with acquired resistance to anti-PD1 blockade in melanoma,^41^ upregulation of other genes in the antigen processing pathway may alter the neoantigen landscapes that results in immune evasion. Alternatively, these upregulated genes may be derived from the residual immune cells, possibly consisting of MDSCs and Tregs, in the relapsed tumors.

We recognize that our proof-of-concept study, with limited number of patient-derived samples and without clinical outcome information, was not sufficient to predict clinical responses to ICB therapies tested here. We envision that a more comprehensive preclinical platform that incorporates establishing autologous materials for genetic and molecular profiling, preclinical testing in humanized mouse models, mechanistic studies and implementation of a parallel clinical trial testing the same agents will allow for improved correlation of preclinical and clinical results and further validation of the utility of this model. These efforts will facilitate development of translatable treatment strategies through preclinical testing that can be rapidly moved to the clinic as rational combination therapies, whereby improved treatment outcomes can be achieved.
