# Supplementary material for: Fidelity of human ovarian cancer patient-derived xenografts in a partially humanized mouse model for preclinical testing of immunotherapies
Source: J Immunother Cancer. 2020 Nov 11;8(2):e001237. doi: 10.1136/jitc-2020-001237 (PMC7661374; doi:10.1136/jitc-2020-001237)
Supplement: Supplementary data [file jitc-2020-001237supp001.pdf]

SUPPLEMENTARY METHODS

Preparing TILs and TALs, and expanding T cells

Tumor-infiltrated leukocytes (TILs) and tumor-associated leukocytes (TALs) were freshly isolated from tumor tissues and/or ascites fluid, respectively. Solid tumor specimens were dissociated using gentleMACS Dissociator (Miltenyi). Mononuclear cells were enriched by density gradient using lymphocyte separation medium. The resulting TILs or TALs contain mixed viable immune cells and tumor cells. HLA2-A2 positivity of the TILs/TALs was determined using flow cytometry. Autologous PBMCs, TALs or TILs (1-2 x 10*6 cells) were seeded in a single well of a 6-well-plate and cultured in AIMV-1640 medium (50% AIM-V, Gibco; 50% RPMI1640; 5% human serum, Gemini Bio-products) and IL-2 (200 U/ml, PeproTech) supplemented with anti-CD3 (OKT3, 1 μg/ml, Bio X Cell) and PHA (1 μg/ml) for 3 days and then expanded for 12-16 days. Floating cells were collected every 3-days and frozen for later use. Cultures were replaced with new medium containing IL2, for 3-4 times.

Establishing PDXs, spheroids, cell lines and tumor measurement

To establish PDXs, 50 μl of minced tumor slurry was IP injected or mixed with 50 μl of Matrigel (BD Bioscience) for SQ injection. Tumor formation were monitored weekly by measuring the abdominal circumference (IP) or using calipers (SQ). Mice were euthanized when the abdominal circumference of the IP tumor reached 9-10 cm and the SQ tumor reach 1500 mm^3, using the reported formula (length x $wide^2/2$). Ascites, peritoneal wash and any visible tumor nodules in the peritoneal cavity from the IP mice were minced and 50 μl of tumor slurry was used to passage the PDXs. In some experiments with treatment, tumor growth was monitored by bioluminescence using IVIS Spectrum (PerkinElmer). For generating spheroids, TILs or TALs were cultured in ultra-low attachment plates (Corning) supplemented with spheroid growth medium, that was composed of serum-free DMEM/F12, human recombinant epidermal growth factor (EGF, 10 ng/mL;

Invitrogen), basic fibroblast growth factor (bFGF,10 ng/mL; Invitrogen), B-27 (Stemcell Technologies), leukemia inhibitory factor (LIF, 5 ng/ml, Sigma), and insulin (10 μg/ml, Sigma), for approximately 2-3 months as described previously [1]. To establish tumor cell lines (from TILs or TALs), cells were cultured in RPMI1640-complete medium which contains 10% fetal bovine serum, 100 U/mL penicillin, 100 μg/mL streptomycin, 2 mM Glutamate, 100 μM sodium pyruvate, 100 μM non-essential minimum amino acid, 20 mM HEPES, and 50 μM 2-mercaptoethanol. The culture was passaged when confluent until it could be passaged once every week (more than 30 generation). The resulting cell culture was deemed to be a cell line (PD-CL).

Both spheroid lines and cell lines were tested to be tumorigenic as they formed progressive SQ tumors (>1cm in diameter) or readily apparent peritoneal tumors/ascites in NSG mice around 2-month post tumor implantation (**figure S2**).

Adoptive T cell transfer and checkpoint blockade antibody treatment

For ACT, IL2-expanded autologous PBMCs or TALs or non-expanded TALs were IP injected into mice at day 14, or 34 (as indicated in the figures) following tumor implantation. Mice were treated with IL2 (2000 U) daily for three days and anti-human blocking antibodies anti-PD1 (200 ug/dose), anti-CTLA4 (100 μg/dose), both anti-PD-1/CTLA4, or control IgG (200 μg/dose) were administered together with IL2 (IP) every 2-3 days for 7 doses. IL2 was given once per week thereafter until mice reached experimental endpoint. All blocking antibodies were from Bio X Cell.

RNA isolation and RNA-sequencing:

RNA was prepared using Tri-Reagent (Sigma) and RNA cleanup kit (Norgen). RNA libraries (TruSeq RNA Library Prep kit, Illumina) were prepared and ran on the Illumina HiSeq 2500 by the RPCCC Genomic Share Resource. Sample reads were aligned to the human reference genome (GRCh38) and GENCODE (version 25) annotation database using TopHat2 [28]. Gene level raw counts were obtained using Subread package [2]. Differential gene expression analysis was

performed using DESeq2 [3] and pathway analysis was performed with the Gene Set Enrichment Analysis (GSEA) method (3.0) [4]. The pre-ranked tool was chosen to run the analysis using a ranked gene list obtained from specific statistical comparisons ran on DESeq2. Pathway analysis was run against MSigDB, a collection of annotated and curated gene set repositories offered by the developer of GSEA (Broad Institute MIT and Harvard). This particular run used C2 of version 7.0 collection, containing 1329 gene sets from various well-known and up-to-date pathway databases such as BioCarta, KEGG and Reactome among others.

Supplementary Table 2: Antibodies used for flow cytometry staining of tumor single cell suspensions.

Antigen	Antibody clone	Supplier	Catalog number	Fluorophore
CD45	H130	BioLegend	304022	Pacific Blue
CD3	OKT3	BioLegend	317307	PE
CD3	HIT3a	BioLegend	300313	APC
CD8a	HIT8a	BioLegend	300920	AF700
CD4	OKT4	BioLegend	317408	FITC
PD-1	EH12.2H7	BioLegend	329920	BV421
TIM3	F38-2E2	BioLegend	345011	APC
LAG3	11C3C65	BioLegend	369306	PE
LAG3	17B4	R&D Systems	FBA2319P	PE
4-1BB	4B4-1	BioLegend	309804	PE
TIGIT	A15153G	BioLegend	372705	APC
CD25	HI30	BioLegend	304012	APC
FoxP3	236A1E7	eBioscience	124777-42	PE
PD-L1	29E2A3	BioLegend	329705	PE
CD48	BI40	BioLegend	336706	FITC
CD80	2D10	BioLegend	305221	BV421
CD86	IT2.2	BioLegend	305421	PE/Cy7
HVEM	122	BioLegend	318807	APC
Galectin 3	M3/38	BioLegend	125410	AF488
Galectin 9	9MI-3	BioLegend	348905	PE
EpCAM	9C4	BioLegend	324220	BV421
E-Cadherin		BioLegend		

HLA-DR	L234	BioLegend	307643	BV-711
CD33	P67.6	BioLegend	366607	PE
CD11b	1CRF44	BioLegend	301329	FITC
CD15	HI98	BioLegend	301907	APC
CD14	63O3	BioLegend	367111	PE/Cy7
CD56	5.1H11	BioLegend	362537	BV-605
LIVE/DEAD fixable Near-IR		Thermo Fisher Scientific	L34975	

1. Liao J, Qian F, Tchabo N, Mhawech-Fauceglia P, Beck A, Qian Z, et al. Ovarian cancer spheroid cells with stem cell-like properties contribute to tumor generation, metastasis and chemotherapy resistance through hypoxia-resistant metabolism. PLoS One. 2014;9(1):e84941. doi: 10.1371/journal.pone.0084941. PubMed PMID: 24409314; PubMed Central PMCID: PMCPMC3883678.
2. Liao Y, Smyth GK, Shi W. The Subread aligner: fast, accurate and scalable read mapping by seed-and-vote. Nucleic Acids Res. 2013;41(10):e108. doi: 10.1093/nar/gkt214. PubMed PMID: 23558742; PubMed Central PMCID: PMCPMC3664803.
3. Love MI, Huber W, Anders S. Moderated estimation of fold change and dispersion for RNA-seq data with DESeq2. Genome Biol. 2014;15(12):550. doi: 10.1186/s13059-014-0550-8. PubMed PMID: 25516281; PubMed Central PMCID: PMCPMC4302049.
4. Subramanian A, Tamayo P, Mootha VK, Mukherjee S, Ebert BL, Gillette MA, et al. Gene set enrichment analysis: a knowledge-based approach for interpreting genome-wide expression profiles. Proc Natl Acad Sci U S A. 2005;102(43):15545-50. doi: 10.1073/pnas.0506580102. PubMed PMID: 16199517; PubMed Central PMCID: PMCPMC1239896.
