# Supplementary material for: Fidelity of human ovarian cancer patient-derived xenografts in a partially humanized mouse model for preclinical testing of immunotherapies
Source: J Immunother Cancer. 2020 Nov 11;8(2):e001237. doi: 10.1136/jitc-2020-001237 (PMC7661374; doi:10.1136/jitc-2020-001237)
Supplement: Supplementary data [file jitc-2020-001237supp002.pdf]

Supplementary Table 1. Patient characteristics

ID	Primary tumor site	FIGO stage	Initial Platinum Status	OCAC Tumor histology
099*	Primary Peritoneal	IIIC	Sensitive	serous
887*	Ovary	IIIC	Refractory	clear cell
842*	Ovary	IIIC	Sensitive	serous
855*	Ovary	IIIA	NA	other
250*	Ovary	IIIC	Refractory	mixed cell
362*	Fallopian	IIIC	Refractory	serous
338*	Ovary	IC1	Sensitive	serous
344*	Ovary	IIIC	Sensitive	serous
145*	Primary Peritoneal	IIIC	Sensitive	serous
998*	Ovary	IIIC	Sensitive	serous
147	Ovary	IA	Sensitive	serous
595	Ovary	IIB	NA	endometrioid
543	Fallopian	IIA	Sensitive	serous
845	Ovary	IA	NA	Nonepithelial
966	Ovary	IIIC	Sensitive	serous
908	Ovary	IA	NA	mucinous
591	Ovary	IVB	Sensitive	serous
787	Ovary	IVB	Resistant	mixed cell
535	Primary Peritoneal	IIIC	Sensitive	serous
342	Ovary	IVB	Sensitive	serous
982	Ovary	IIC	Sensitive	serous

* indicate patient's' samples used for generating experimental models in this study.

Figure S1

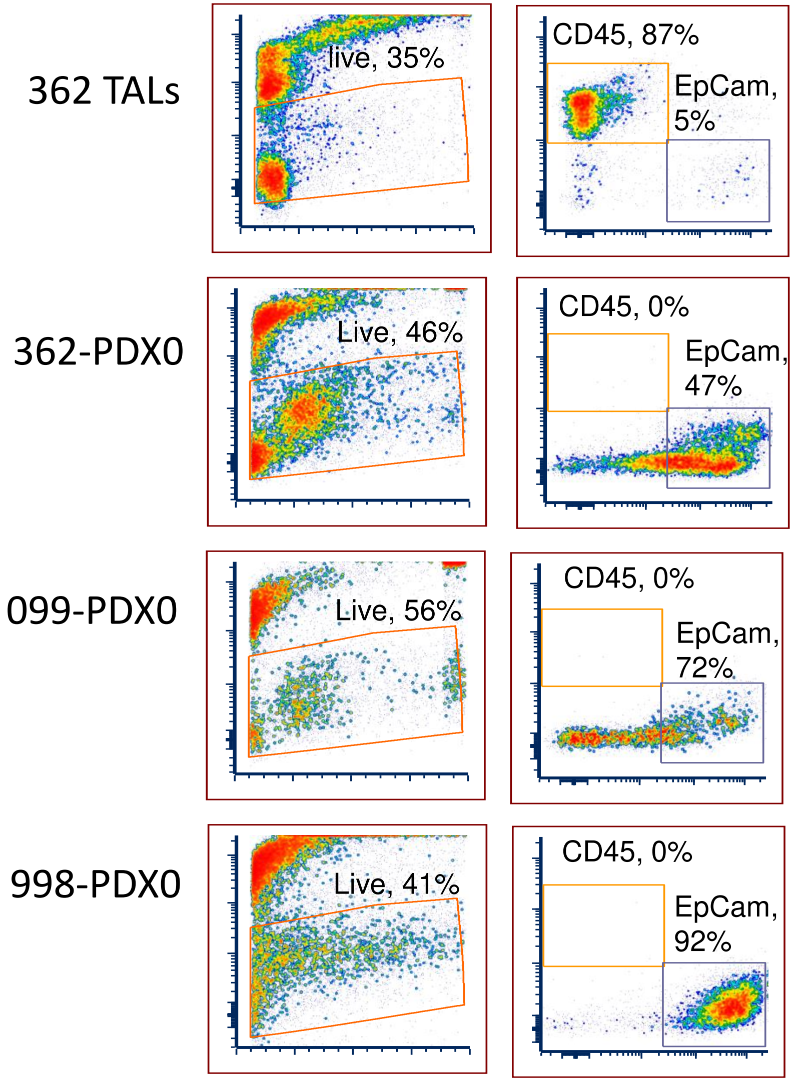

Figure S1. Immune cells are not present in the PDX0 generated from patient-derived TIL/TALs. Representative flow cytometry plots of one original TALs and 3 PDXs. (A) One of the original TALs sample used for establishing PDXs. (B) 362-PDX0, (C) 099-PDX0, (D) 998-PDX0. Flow plots were gated for live cells (left panels and then gated for CD45 and EpCAM positive populations. Percentage of each population is shown.

Figure S2

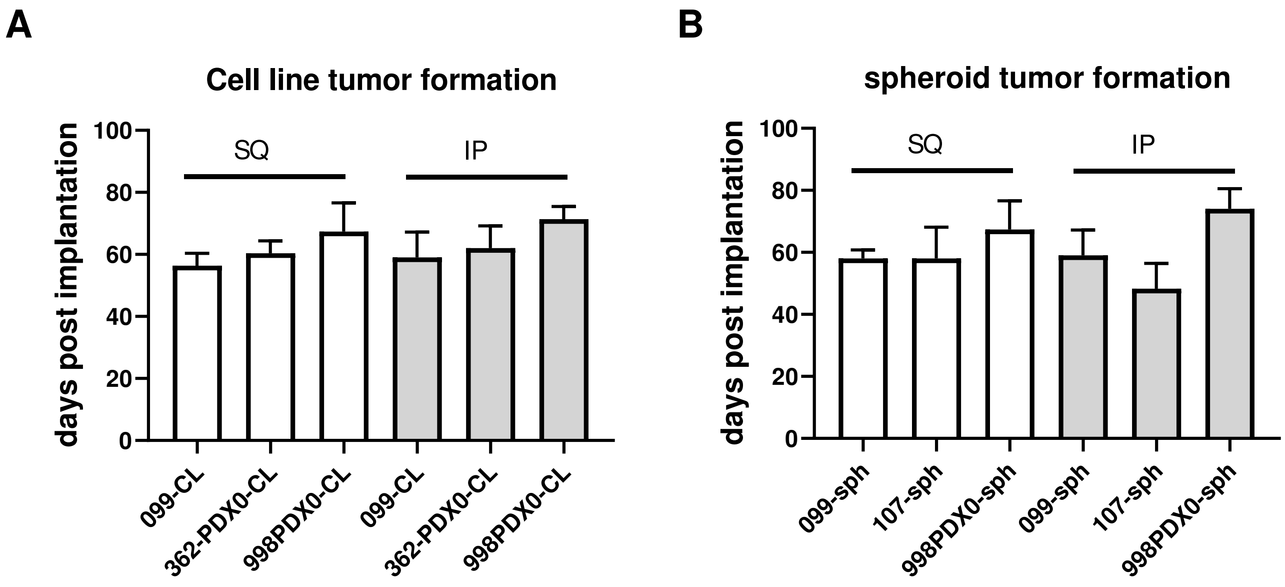

Figure S2. Tumorigenicity of PD-CLs and PD-sphs derived from human OVC specimens. (A) Days of tumor formation of 3 PD-CLs. (B) Days of tumor formation of 3 PD-sphs. Approximately 1 x 10^6 (SQ) or 2 x 10^6 (IP) cells of PD-CLs and PD-sphs (1 x 10^5) were injected into each mouse. Graphs represent data from 2-4 mice measured (days post tumor implantation) when subcutaneous tumors reach ~1 cm in diameter (SQ) or abdominal circumference at ~8 cm for intraperitoneal tumors.

Figure S3

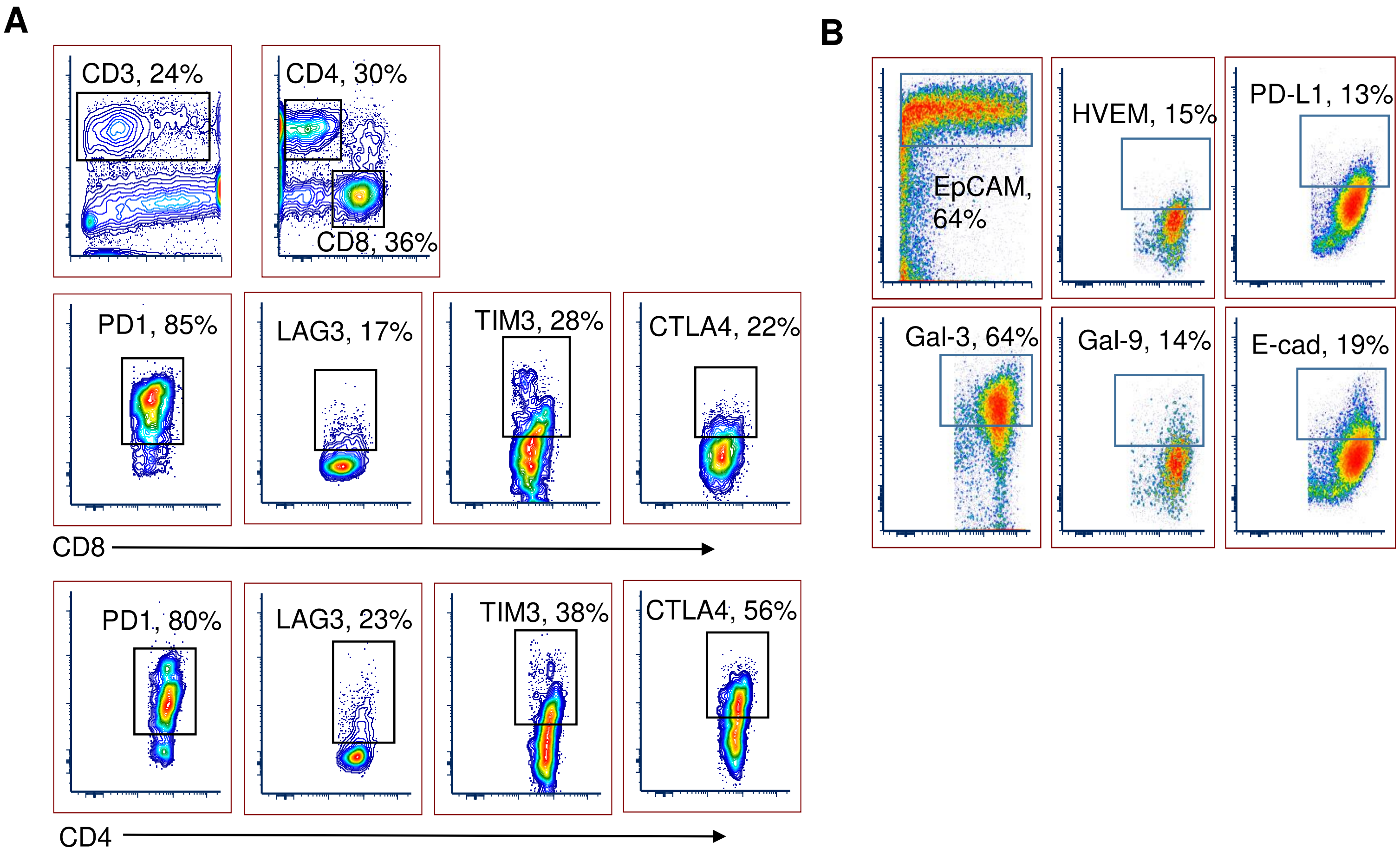

Figure S3. Examples of flow cytometry plots of immune checkpoints and their ligands in human ovarian tumor microenvironment. (A) Expression levels of exhaustion makers, PD1, LAG3, TIM3, and CTLA4 on CD8 and CD4 T cell surface. (B) Expression levels of ligands HVEM, PD-L1, Gal-3, Gal-9, and E-Cadherin in EpCAM+ cells. Gating was based on FMO (fluorescence minus one) staining.

Figure S4

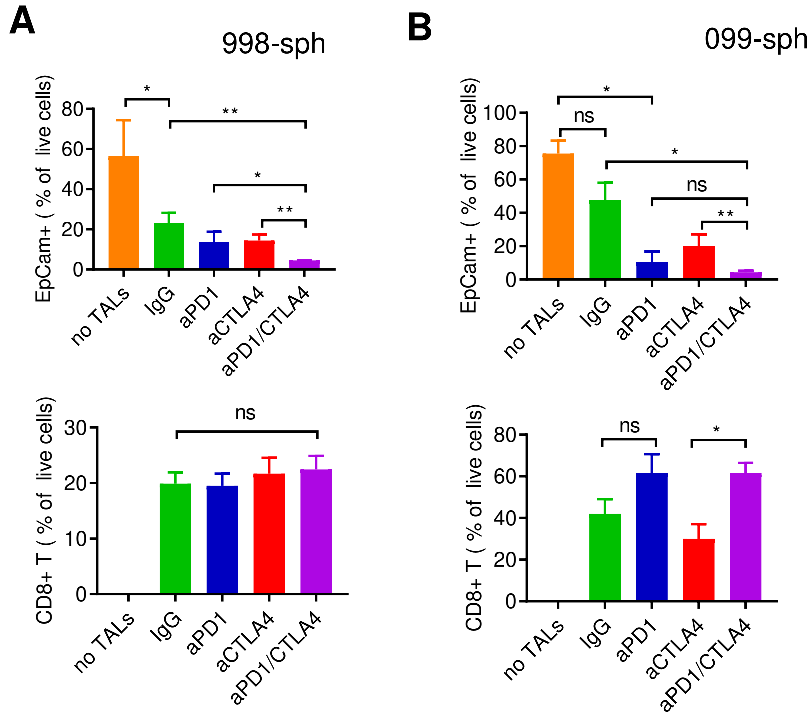

Figure S4. Checkpoint blockade in coculture of IL2-expanded TALs and PD-sphs. (A) 998-sph, (B) 099-sph. Percentage of remaining EpCAM+ sph cells (top panel) and CD8+ T cells (bottom panel). IL2-expanded autologous TALs (~1 x 10^5 in A and 5 x 10^4 cells in B) were mixed with PD-sphs (approximately equal number of cells) in spheroid growth medium and treated with IgG (16 μg/ml), anti-PD1 (15 μg/ml), anti-CTLA4 (16 μg/ml), or both anti-PD1 and anti-CTLA4 for 9 days in the presence of IL2 (100 U/ml). Medium with IL2 and antibodies were replaced at day 5. Cells were stained with antibodies for EpCAM, CD3, CD4, and CD8 populations. Percentage of gated cell population was analyzed. Data shown are mean±SD of triplicate wells and are representative of duplicated experiments. Statistical significance is indicated as follows: *, p<0.01, **, p<0.005, ns, not significant (student's t test, two tailed and unpaired).

Figure S5

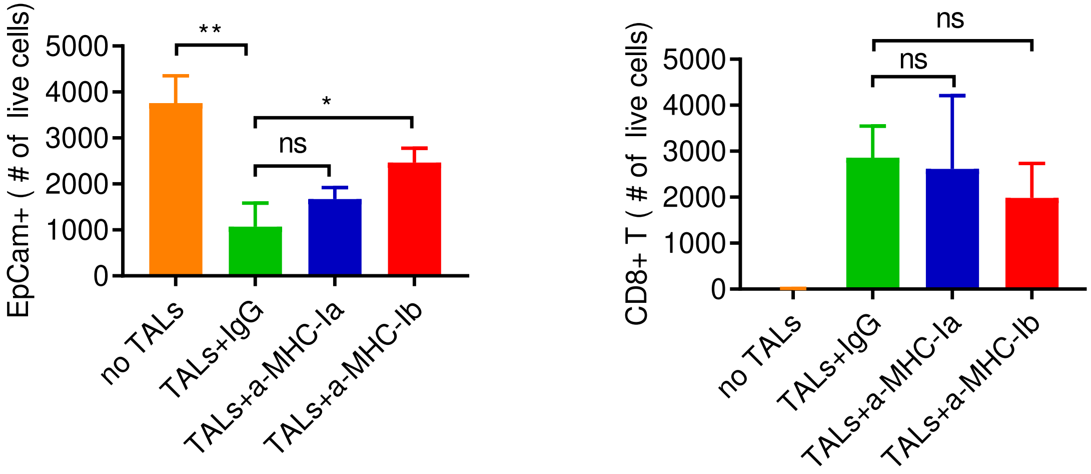

Figure S5. Blockade of MHC-class I (HLA-A, HLA-B, HLA-C) rescue T cell killing of EpCAM+ PD-spheroids in vitro. Percentage of remaining EpCAM+ sph cells (A) and CD8+ T cells (B) is shown. IL2-expanded autologous TALs (~5 x 10^4 cells) were mixed with 099-PDX0 sphs (~5 x 10^4 cells) in spheroid growth medium and treated with mouse IgG2a (20 μg/ml), or pan anti-MHC-class I antibody (20 μg/ml, a-MHC-Ia, or 40 μg/ml, a-MHC-Ib; clone W6/32 from Bio X Cell), for 9 days in the presence of IL2 (100 U/ml). Medium with IL2 and antibodies were replaced at day 5. Cells were stained with antibodies for EpCAM, CD3, CD4, and CD8 populations. Live events of gated cell population were analyzed. Data shown are mean±SD of three different wells and representative of 2 independent experiments. Statistical significance is indicated as follows: *, p<0.01, **, p<0.005, ns, not significant (student’s t test, two tailed and unpaired).

Figure S6

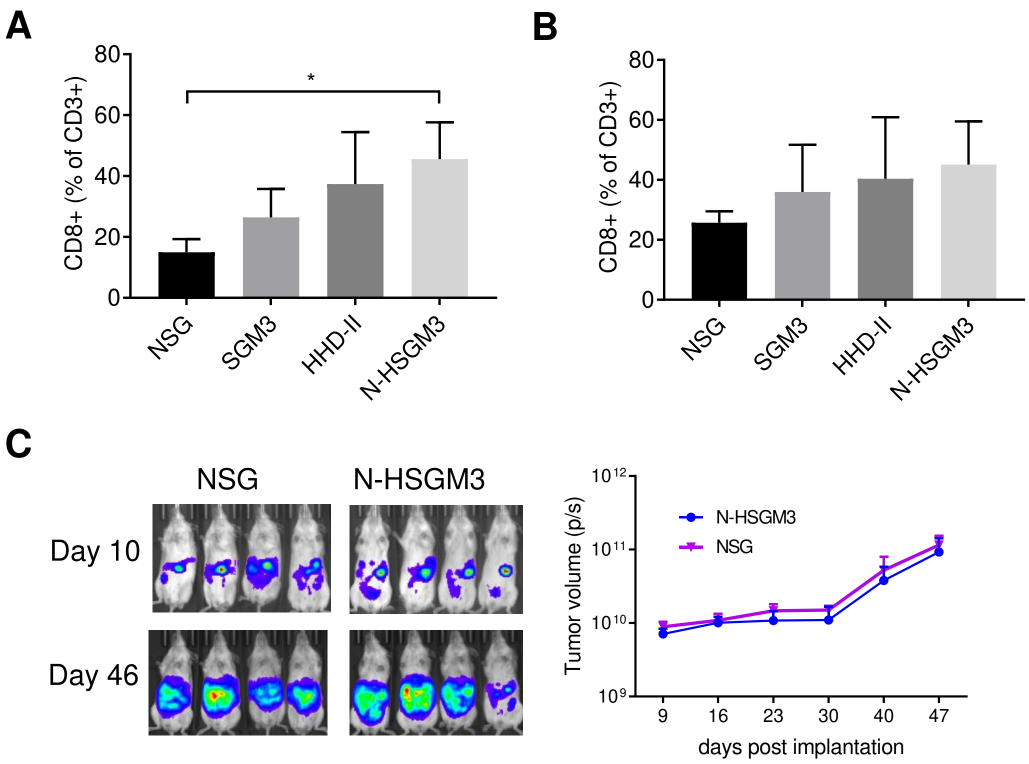

Figure S6. PBMC and tumor engraftment in various NSG sub-strains. (A-B) Engraftment efficiency of normal PBMC in NSG and its sub-strains HHD-II, SGM3 and N-HSGM3 mice (n=3). The frequency of CD8+ T cells in the blood (A) and spleen (B) is trended higher in the N-HSGM3 than the NSG mice at day 60 post infusion. (C) Similar engraftment efficiency of PD-CL in NSG and N-HSGM3 mice. NSG mice (wild-type) or heterozygous for HHD and SGM3 were injected (IP) with 099-CL-Luc cells. Tumor load was assessed by bioluminescent imaging (p/s) of 099-CL-Luc OVC tumor-bearing mice at indicated time points post implantation.

Figure S7

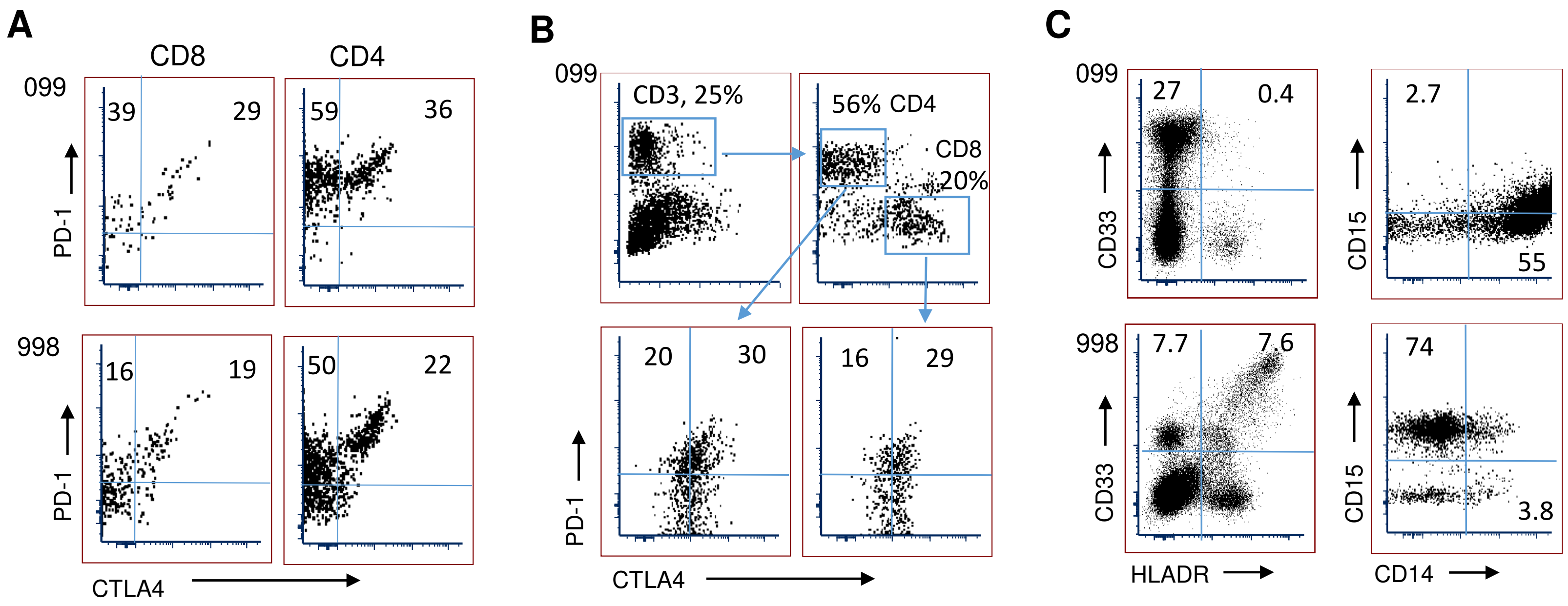

Figure S7. Immune checkpoint expression on IL2-expanded or non-expanded TALs and MDSC population in human OVC specimens. (A) Immune checkpoint expression on IL2-expanded 099 and 998 TALs. Numbers represent percentage of cells positive for immune checkpoints PD1 and/or CTLA4 on CD8 and CD4 T cell. TALs from ascites were cultured in IL2 (200 U/ml) for 12 days and stained for CD3, CD8, CD4, PD1 and CTLA4 surface expression. (B) Immune checkpoint expression on non-expanded TALs (099). TALs were thawed in IL2 (100 U/ml) for 16 hours and stained for CD3, CD8, CD4, PD1 and CTLA4 surface expression. (C) Representative FACS plots depicting myeloid population in 099 and 998 TALs. Cells from each sample were stained with antibodies against CD45, HLADR (DR), CD33, CD11b, CD14, and CD15 and analyzed using flow cytometry. Numbers represent percentage of cells positive for the gated population.

Figure S8

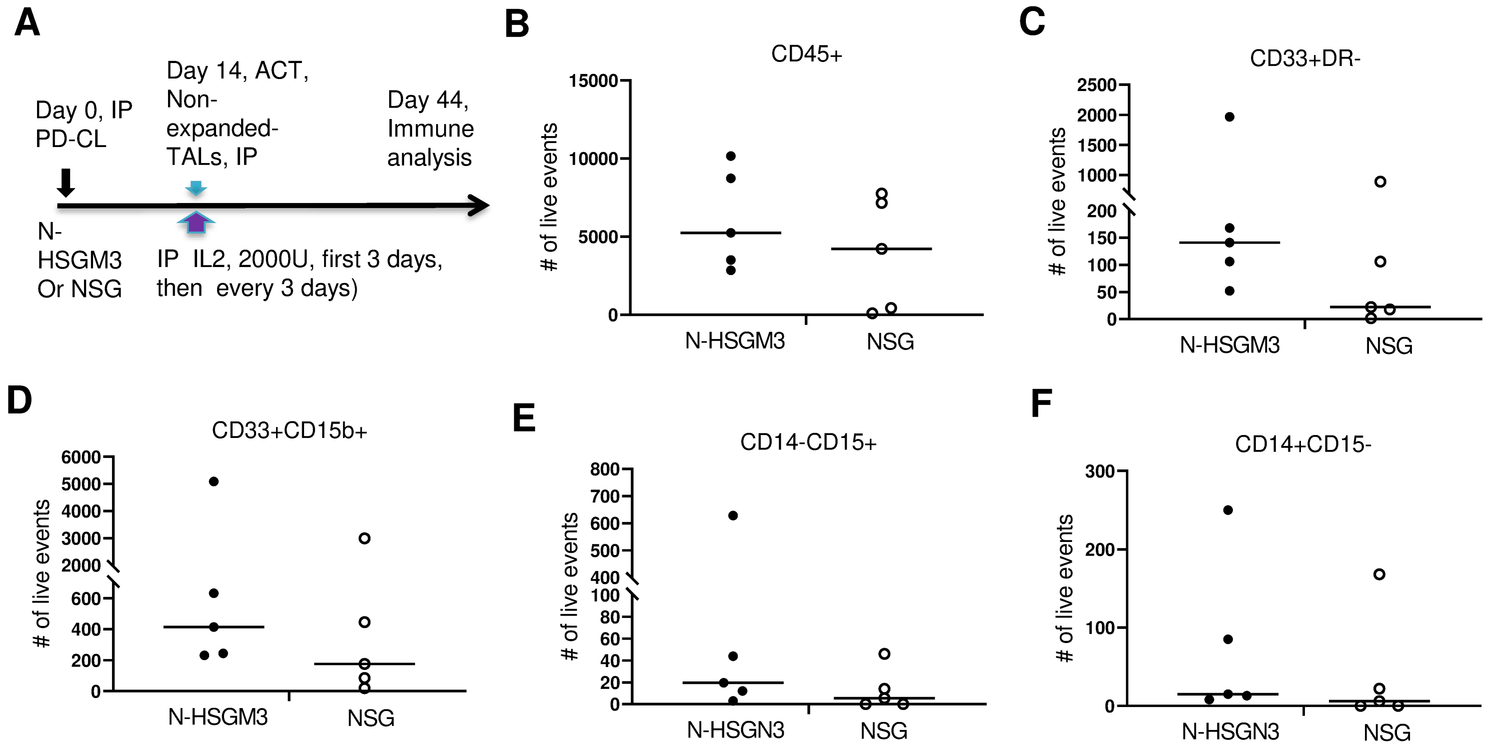

Figure S8. Engraftments of MDSC population in NSG and N-HSGM3 strains. (A) Experimental scheme for tumor implantation and adoptive cell transfer (ACT). Mice were injected with 1.6 x 10* 6 cells and infused with unexpanded autologous TALs (3 x 10*5) and IL2 at indicated time. Flow cytometry data showing the live population of (B) CD45+, (C) CD33+HLADR-, (D) CD33+CD11+, (E) CD14-CD15+, and (F) CD14+CD15- cells in the peritoneal wash of tumor-bearing mice. NSG and NHSGM3 mice were injected (IP) with 099-PDCL-Luc cells. Tumor formation was confirmed by bioluminescent imaging (not shown). The numbers of total CD45+ cells and different MDSC populations in the peritoneal wash were mostly trended higher in the N-HSGM3 than the NSG mice at day 32 post infusion, albeit not statistically significant.

Figure S9

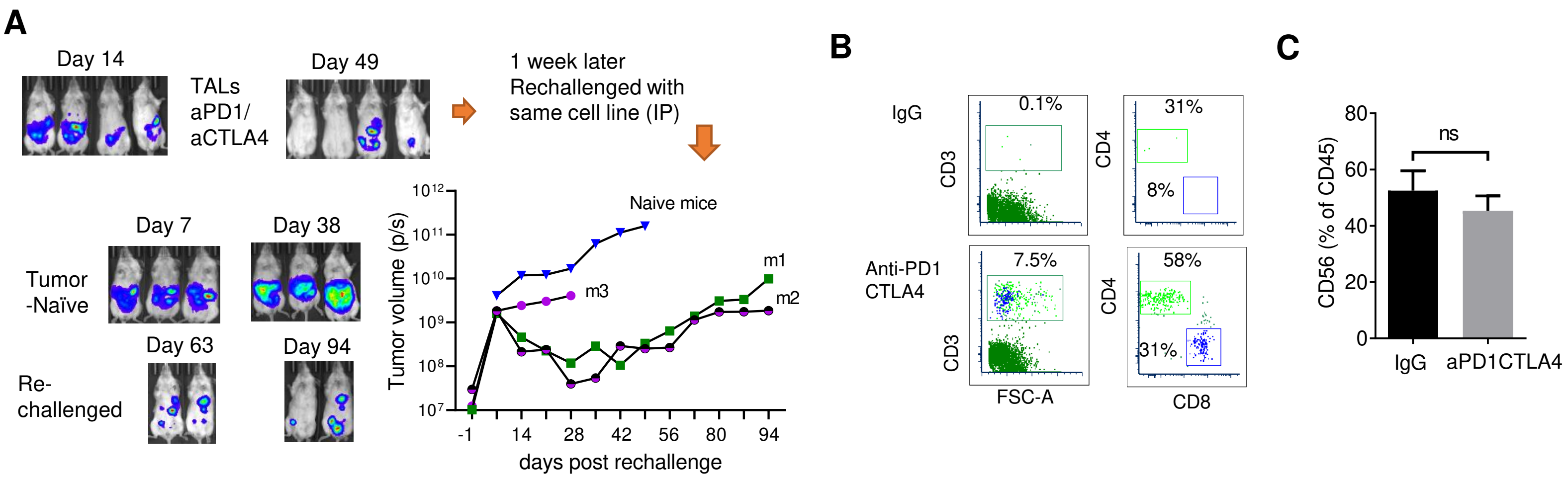

Figure S9. Antitumor memory response in mice with minimal tumors after initial ICB. (A) Persistence of the remaining T cells in the dual anti-PD1/CTLA4 blockade responder mice. Initial treatment was the same as in **figure 5A**, tumor-free mice (n=3) from the dual anti-PD1/CTLA4 blockade group were rechallenged (IP) with the same number of 099-Luc-CLs at day 56. Tumor-naïve mice (n=3) were also injected with the same number of cells and served as controls. Tumor growth were monitored using bioluminescent imaging for additional 50-90 days. Images shown are initial tumor load of dual blockade treated mice at day 14 and day 49 (top panels), and tumor load of tumor-naïve and rechallenged mice at indicated day post initial tumor implantation (bottom panels). Days after post tumor inoculation of the rechallenged mice are shown in the luminescent volume graph (right panel). One of the rechallenged mice developed earlier progressive disease. (B) Representative flow plots of T cell composition in PW of mice treated with IgG or dual ICB. Numbers represent percentage of cells positive for the gated population. (C) Frequency of total CD56+ cells in the CD45+ population in the PW of IgG and dual ICB treated mice. ns, not significant.

Figure S10

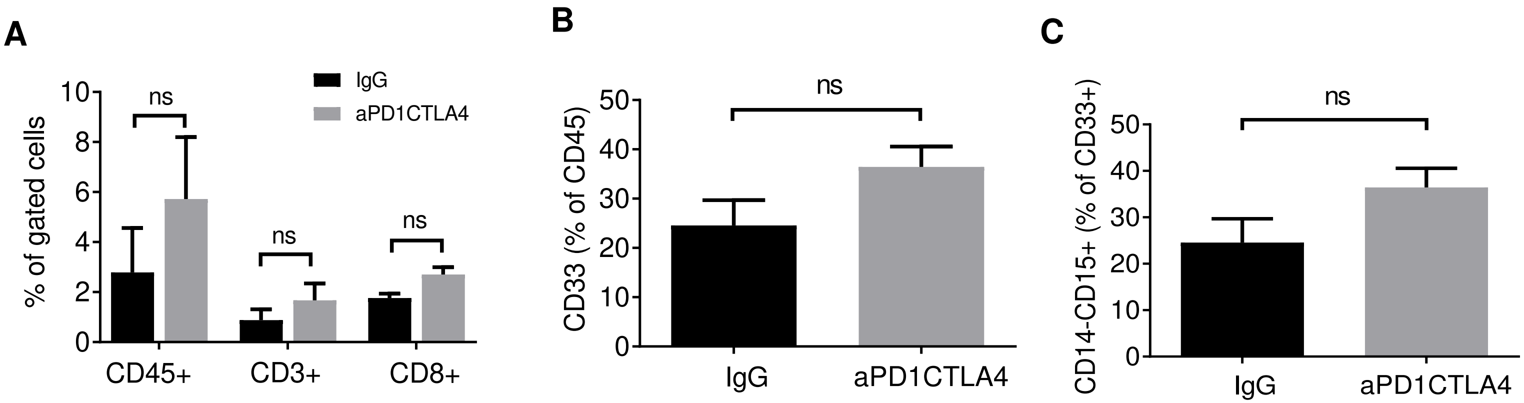

Figure S10. Dual ICB mildly increased CD3+CD8+ T cells and MDSC cells in the peritoneal wash of mice implanted with 998-PDX1-sph and autologous TALs at end point. (A) Frequencies of CD45+, CD3+ and CD8+ T cells, (B) Frequency of CD33+ MDSC, (C) Frequency of CD14-CD15+ MDSC. Live cells from the peritoneal wash samples from the IgG- and dual aPD1/CTLA4-treated mice (from **figure 5C-D**) were stained with antibodies specific for CD45, CD3, and CD8 for T cells, or CD45, CD33, CD11b, CD14, and CD15 for MDSC cells. ns, not significant using unpaired, two-tail t test.

Figure S11

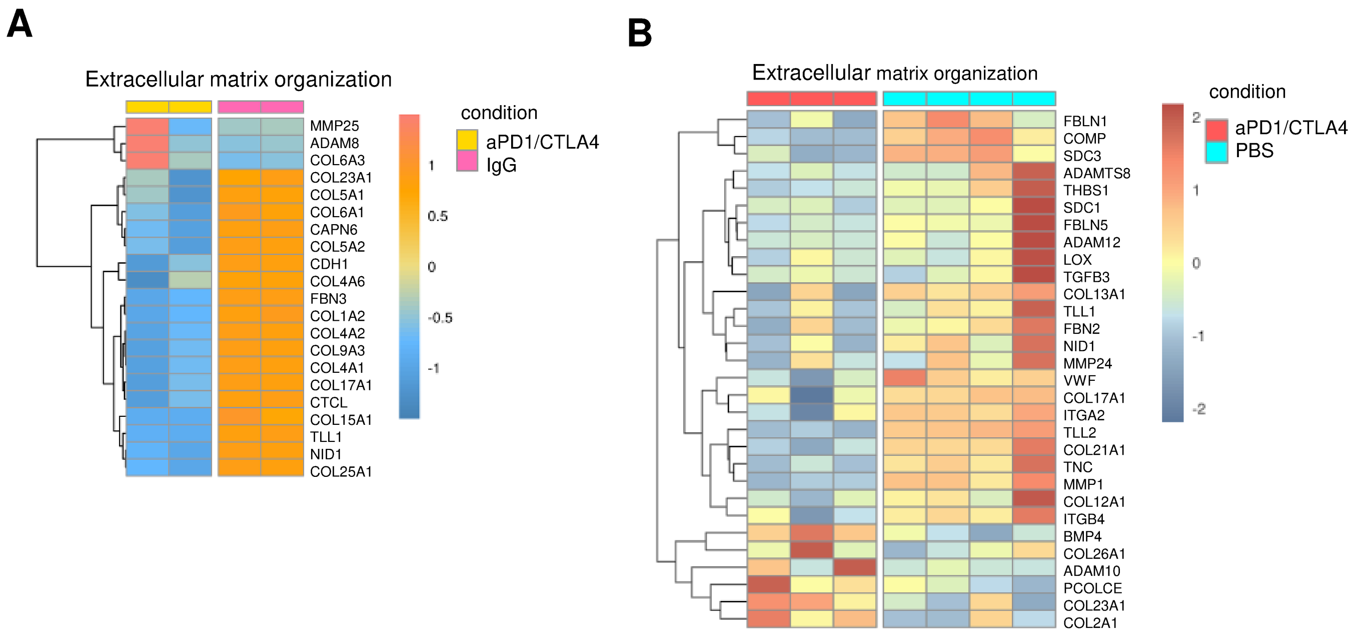

Figure S11. Transcriptome changes in OVC TME in N-HSGM3 mice treated with or without dual anti-PD1/CTLA4 antibody blockade combined with ACT. (A) Representative heatmap showing one of the down-regulated pathways from the GSEA of peritoneal wash (PW) samples from mice treated with anti-PD1/CTLA4 antibodies relative to those with IgG. (B) Representative heatmap showing one of the down-regulated pathways from the GSEA of tumors from mice treated with TALs plus anti-PD1/CTLA4 antibodies relative to those with IgG PBS. DE analysis performed at 2 fold changes using FDR<0.05.
